# A systemic risk assessment methodological framework for the global polycrisis

**DOI:** 10.1038/s41467-025-62029-w

**Published:** 2025-08-14

**Authors:** Ajay Gambhir, Michael J. Albert, Sylvanus S. P. Doe, Jonathan F. Donges, Nadim Farajalla, Leandro L. Giatti, Haripriya Gundimeda, Sarah Hendel-Blackford, Thomas Homer-Dixon, Daniel Hoyer, Sumaya Adan, David Jacome-Polit, Luke Kemp, David Korowicz, Zora Kovacic, Jan Kwakkel, Laurie Laybourn, Robert Lempert, Ayan Mahamoud, Tom H. Oliver, Ivana E. Pavkova, Joseph Ponnoly, Vishwas Satgar, Megan Shipman, Jana Sillmann, Nick Silver, Samuel Stevenson, Ruth Richardson

**Affiliations:** 1 Accelerator for Systemic Risk Assessment (ASRA), Washington, DC USA; 2 Grantham Institute, Imperial College London, London, UK; 3 School of Social and Political Science, University of Edinburgh, Edinburgh, UK; 4 GeoSustainability Consulting, Adabraka-Accra, Ghana; 5 Earth Resilience Science Unit, Potsdam Institute for Climate Impact Research, Member of the Leibniz Association, Potsdam, Germany; 6 Lebanese American University, Chouran, Beirut Lebanon; 7 School of Public Health, University of São Paulo, São Paulo, Brazil; 8 Department of Economics, Indian Institute of Technology Bombay, Mumbai, Maharashtra India; 9 Cascade Institute, Royal Roads University, Victoria, BC Canada; 10 Complexity Science Hub. Vienna, Vienna, Austria; 11 Societal Dynamics (SoDy), Toronto, Canada; 12 Oxford Martin School, University of Oxford, Oxford, UK; 13 ICLEI, Bonn, Germany; 14 TU Delft, Delft, The Netherlands; 15 Centre for the Study of Existential Risk, Cambridge, UK; 16 Korowicz Human Systems, Balcarrick, Donabate, Co. Dublin, Ireland; 17 Urban Transformation and Global Change (TURBA) Lab, Universitat Oberta de Catalunya, Barcelona, Spain; 18 Faculty of Technology, Policy and Management, TU Delft, Delft, The Netherlands; 19 Global Systems Institute, University of Exeter, Exeter, UK; 20 Frederick S. Pardee Center for Longer Range Global Policy and the Future Human Condition, RAND Corporation, Santa Monica, CA USA; 21 School of Biological Sciences, University of Reading, Reading, UK; 22 TMP Climate, Tokyo, Japan; 23 Cinfodens Consulting, Pearland, TX USA; 24 School of Social Sciences, University of Witwatersrand, Wits, Johannesburg, South Africa; 25 Center for International Climate Research, Oslo, Norway; 26 Research Unit Sustainability and Climate Risks, University of Hamburg, Hamburg, Germany; 27 Bayes Business School, London, UK

**Keywords:** Sustainability, Research management

## Abstract

Human societies and ecological systems face increasingly severe risks, stemming from crossing planetary boundaries, worsening inequality, rising geo-political tensions, and new technologies. In an interconnected world, these risks can exacerbate each-other, creating systemic risks, which must be thoroughly assessed and responded to. Recent years have seen the emergence of analytical frameworks designed specifically for, or applicable to, systemic risk assessment, adding to the multitude of tools and models for analysing and simulating different systems. By assessing two recent global food and energy systemic crises, we propose a methodological framework applicable to assessing systemic risks in a polycrisis context, drawing from and building on existing approaches. Our framework’s polycrisis-specific features include: exploring system architectures including their objectives and political economy; consideration of transformational responses away from risks; and cross-cutting practices including consideration of non-human life, trans-disciplinarity, and diversity, transparency and communication of uncertainty around data, evidence and methods.

## Abstract (web summary)

This paper proposes a framework to assess systemic risks that compound and cascade within and between systems. This emphasizes political economy and transformations, as well as trans-disciplinarity and diverse participation, evidence and methods.

## Introduction

The world is facing multiple, interconnected risks, many of which have materialised into what has been called a global ‘polycrisis’^1^. Recent crises touching many systems (finance, economy, food, energy, health, security and more besides) include the 2007–2009 Global Financial Crisis, 2020–2022 COVID-19 pandemic, 2022 Russia invasion of Ukraine and 2023 Israel-Hamas conflict, with the latter two cases reflecting a period of rising geo-political tensions. These have occurred in the context of climate change driving more severe and frequent extreme weather events^2^, transgression of an increasing number of safe planetary boundaries for biophysical and biochemical processes^3^ and severe biodiversity loss that has been termed a ‘mutilation of the tree of life’^4^. Since 1990 inequality has grown within countries representing almost three-quarters of the global population, eroding trust and destabilising political systems^5^, whilst technology-related risks including cyber-security, misinformation and artificial intelligence (AI) rank highly on risk surveys (e.g. ref. ^6^). The interconnections between these risks, their geographical reach and ability to exacerbate one-another mean we are in a world of global systemic risk, which has been asserted as more serious, in terms of scale and danger, than risks seen before^7^.

Systemic risks are those that can affect a whole system (e.g. a financial system, nationally, regionally, or even globally), as opposed to risks confined to a single part of it (referred to, most commonly in the financial system, as ‘idiosyncratic’ or firm-specific risks^8^), as well as risks potentially facing many systems at once^9^. While there are many definitions^10^, we conceive of systemic risks as the potential for individual failures to cascade into system-wide breakdown, both within and across systems. The emergence of global systemic risk^11^ in an increasingly environmentally degraded, technologically advanced and interconnected world, has been closely connected to what has been termed our current polycrisis of ‘causal entanglement of crises in multiple global systems’^9^. Many other concepts, such as complex adaptive systems, interactions within systems of systems and multi-hazard risks, are relevant to the concepts of global systemic risk and the polycrisis (see Supplementary Table 1). Whilst definitions are not firm, their general features are shared: multiple, interconnected risks which can do considerable harm to humanity and ecosystems.

Risk management and analysis are well-established practices when applied to single issues and threats, or to particular organisations. However, the practice of systemic risk assessment—and the design of systemic risk responses—is much newer, at least outside of the financial sector where, especially since the 2007–2009 Global Financial Crisis, many analytical models and frameworks of contagion through networks have been proposed^12^. Calls to understand interconnections and feedbacks between different systems’ risks include around biodiversity loss and climate extremes^13^ and ‘synchronous failures’ that climate change could induce by exacerbating vulnerabilities in economic, water, land and food systems^14^. Some inter-systemic risks have already been studied and even quantified, such as feedbacks between financial and real economic systems that could exacerbate financial crises^15^.

Several established and emerging frameworks allow assessment of interacting, systemic risks^16–22^ (Table 1). They contain commonalities: exploring and understanding the system (or systems) including its goals; systems mapping to understand system interconnections, strategy and response development; scenario development; and implementation, monitoring and review or adaptations going forward.

**Table 1:** Existing frameworks to understanding interconnected risks across systems

Framework	Description
**Systemic risk and multi-hazard assessment frameworks**	
Cascade Institute Polycrisis Analysis Framework^16^	Application of causal mapping techniques to capture implications of risks and crises spreading between systems, distinguishing between slow-moving stresses and fast-moving unpredictable trigger events. The Stress-Trigger-Crisis (STC) model, in particular, utilises three steps: (1) Domino diagrams, to depict how stresses combine with triggers to lead to crises and how those elements interact with stresses, triggers and crises across other systems moving forward across time; (2) Inter-systemic feedback diagrams, to capture relationships between stresses, triggers and crises, including feedback loops which show how effects lead back to causes; (3) Analysis and interpretation of these diagrams, to identify the most vulnerable and influential elements, including those that influence dynamics through interaction and pernicious positive feedback loops.
MYRIAD-EU 6-step framework for individual, multi- and systemic risk analysis and management^17^	A framework developed in the EU Horizon project ‘MYRIAD-EU’, to assess systemic risks, consisting of the following stages: (1) Finding a system definition; (2) Characterisation of direct risk; (3) Characterisation of indirect risk; (4) Evaluation of direct and indirect risk; (5) Defining rRisk management options; and (6) Accounting for future system state. This approach is designed to allow for an accounting of risk dynamics, explicit focus on indirect risks, use of multiple lines of evidence, cross-scale analysis and stakeholder engagement and co-production.
Systems Thinking Toolkit for UK Civil Servants^18^	A process to structure thinking about systems, consisting of four iterative steps: (1) Confirm the goal; (2) Understand the system; (3) Co-design and test; and (4) Implement, monitor and evaluate. These steps are supported by guidelines on multiple tools and techniques to help with each stage, including systems mapping, context diagrams and theory of change maps.
SysRisk approach^19^	Process for UK recovery from COVID-19 to understand systemic environmental risk. Involves participatory systems mapping with experts representing a wide range of cognitive diversity (i.e. Political, Economic, Social, Technological, Legal, Environmental domain-specific expertise) to identify ‘risk cascades’ linking long-term drivers to proximate impacts on health and wellbeing. Data/monitoring watchpoints are mapped, in order to track whether different risk cascades are being realised, along with policy intervention points to disrupt risk transmission.
International Risk Governance Council (IRGC) Guidelines on Systemic Risk Governance^20^	Designed specifically to understand systemic risks, using seven interlinked steps: (1) Explore the system; (2) Developing scenarios; (3) Determine goals and levels of risk tolerance; (4) Co-develop risk management strategies; (5) Address unanticipated barriers and sudden shifts; (6) Decide, test and implement strategies; and (7) Monitor, learn, review and adapt.
**Other frameworks applicable to multi-risk assessment**	
Infrastructure resilience stress-testing approach^21^	Moves beyond single-issue, single-component stress-testing for infrastructure, to systemic stress-testing accounting for interconnectedness across systems, using a multi-tiered approach: (1) Screening level assessment to frame critical system functions and analyse response to shocks and stressors; (2) Simple systems modelling to understand and quantify connections across domains and impact on critical system functions; and (3) Advanced modelling (e.g. using AI and network science) to test interconnected network resilience in light of random and targeted threats/attacks.
Decision-making under deep uncertainty (DMDU) methods^22^	A range of methods designed as an alternative to ‘predict-then-act’ measures, so as to better account for uncertainties and the need for adaptiveness. For example, Robust Decision-making (RDM)^63^ tests policies and strategies across a range of different futures, to identify robust, preferred policies that perform the best in a range of futures. Uses a 5-step iterative process: (1) Decision Framing; (2) Evaluate strategies across futures; (3) Vulnerability Analysis; (4) Trade-off Analysis; and (5) New Futures and Strategies. These steps help identify decision-relevant scenarios and robust strategies and are designed to support stakeholder engagement in contested decision environments.

This table does not give an exhaustive overview of all frameworks available, but includes those deemed most relevant by the authors as part of the research and discussion process (as shown in Supplementary Fig. 7).

It is critical to have in place a systemic risk assessment framework that can explicitly address the current global systemic risk landscape and resulting polycrisis, from its deep roots of interconnected social, economic and environmental risks stemming from the ‘Great Acceleration’^23^, including inequality and injustices (within and between human and non-human systems) deriving from colonialism and resource extraction. Here, we propose and develop a methodological framework, taking as our point of departure these frameworks, whilst also considering the numerous (mainly modelling) analytical approaches that are used to simulate particular systems. To do so, we use a process of understanding the key features and dynamics of two historical global systemic crises across coupled food and energy systems (those of 2008 and 2022), to identify how to apply and combine existing approaches to more holistically assess systemic risks across these systems and others.

## Food and energy system entangled crises as a lens for developing a systemic risk framework

In a highly interconnected world where risks have the potential to cascade across systems, regions and scales, systemic risk assessment potentially encompasses a consideration of everything. However, some bounding is required to keep consideration of risks tractable. Here, we start with a specific pair of systems (global food and energy systems) to act as a lens through which to consider a range of systems and their interrelated risks.

Focusing on these two systems and their interconnections is salient for a number of reasons. Energy systems power all socio-economic activity, including input into agriculture^24^. Food systems are vital for addressing hunger and poverty, whilst lower energy poverty is closely correlated with higher health and educational outcomes^25^. Yet more than 2 billion people worldwide are experiencing food insecurity^26^ whilst nearly 800 million people still lack access to electricity (and this number increased in 2022^27^). From a moral, as well as economic and social, standpoint, understanding the risks to food and energy systems is therefore critical. Second, food and energy systems crises are often closely related to other crises, including financial crises, extreme weather events, conflicts as well as disruptions to international and national supply chains^9^. Third, the potential impacts of food and energy systems on other systems is considerable. The global food system is responsible for a third of greenhouse gas emissions^28^, whilst energy use, through the burning of fossil fuels (some of which occur in the food system), is responsible for over 70% of current greenhouse gas emissions^29^. Both systems help drive land use change, water consumption and chemical pollution.

It is briefly worth describing what food and energy systems are, in order that they can be understood in the context of systemic risks. Whilst there is no commonly agreed definition, several descriptions of food systems recognise that they are an interconnected set of links between the factors that go into food production (i.e. the land, ecology, fertiliser, seeds, nutrients, water, labour, machinery), its transport, storage and supply and the local distributors, retailers and consumers of food products^30^. This description immediately highlights that food systems interact with, and are in fact embedded in, other systems, including ecological systems, economic systems, political systems and social systems. Energy systems are defined by the Intergovernmental Panel on Climate Change as the set of components related to the production, conversion, delivery and use of energy^31^. This somewhat anodyne definition avoids any discussion of the actors in the system—and their power, or lack thereof - as it currently stands today, be they fossil fuel producers, end users without reliable electricity access or low-carbon energy technology developers.

A more holistic definition of food and energy systems would explicitly encompass such contexts of power and political economy, which are vital to their operations and functions. Moreover, it would encompass their nature as dynamic, complex adaptive systems^32^, which evolve over time. For example, food systems have seen a shift from diverse food traditions and regional identity towards monotonous diets, industrialisation and ultra-processed foods, with consequences for obesity, undernutrition, climate and biodiversity^33^. Energy systems are going through an accelerating (yet globally still too slow) transition from fossil fuel-dependence towards low-carbon energy technologies, raising issues of justice, incumbency and power^34^. Finally, these systems operate at many scales, from local to global. In this study, the point of focus is the latter, accepting that the ultimate beneficiaries, or losers, from such systems will in many cases be at the former scale.

## Understanding the dynamics of historical food-energy systems crises

A tangible way of developing a systemic risk assessment framework using analysis of entangled energy and food systems is to detail the different factors at play, their interactions and dynamics, as well as the risk mitigation mechanisms, when considering historical crises, using a ‘retrodiction’ analysis.

We conceptualise a crisis as the materialisation of a risk and for the purposes of our study define it as a sudden event - or series of events - that causes considerable harm to the wellbeing of a large number of people, other species or ecosystems, over a relatively short period of time. This builds directly on recent definitions^9^ but develops them to recognise harms beyond those to people, recognising, for example, our current biodiversity loss crisis^4^. We explore two notable ‘crises’ which both gave rise to multi-regional energy and food supply disruptions and price surges, with devastating consequent impacts on food insecurity, energy poverty and a range of knock-on consequences.

Multiple analyses of the entangled elements of the 2008 food-energy crisis^35–38^ identify several interacting factors. Rising energy demand, combined with lowering energy return on energy invested (EROI)^39^ due to a decline in the quality of mature oil field production all combined to lead to a surge in oil prices. This drove food price increases, via increased fertiliser production and energy use costs, as well as incentivising greater production of biofuels as a substitute for oil, with consequent impacts on food production as bio-crops were prioritised. Such forces interacted with other disruptors to food production, including extreme weather and water scarcity affecting major food producing regions including an Australian extended drought, as well as longer term stressors of rising food demand in the context of falling agricultural productivity. Financial speculation and food export restrictions provided further stresses on food supply, increasing volatility and exacerbating food price rises respectively^40^. Although the significance of many factors has been contested^41^, the overall link between the energy price surges and food price surges is well established.

**Fig. 1:**
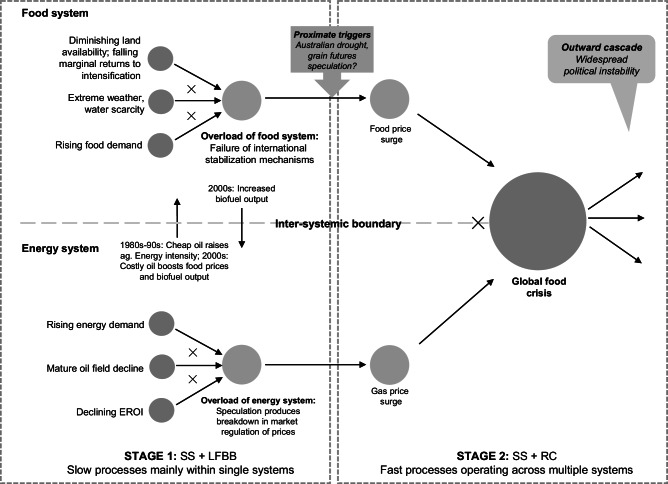
The 2008 global food and energy crisis. This figure depicts (on the left-hand side) long term simultaneous stresses (SS) which have built up over time. It also depicts long-fuse big bang (LFBB) processes, which represent the accumulation of stresses within systems until the systems’ coping capacity is exceeded (system overload), resulting in a sudden, non-linear shift in system behaviour. In the energy system, EROI is energy return on investment, which is the ratio of the energy output from an energy resource to the energy input to obtain that output. The figure shows how long-term stresses in food and energy systems have led to the overload of these systems. It also depicts the interaction between these systems (through the transmission channels of energy input into food prices and of biofuel output from cropland into energy systems) that effectively coupled these systems together. The food system, in its overloaded state, was susceptible to the trigger of the Australian drought, depicted in the middle of the figure, leading to a food price surge. As depicted on the right-hand side of the figure, this food price surge was compounded by a gas price surge from the overloaded energy system, exacerbating the resultant global food crisis. This crisis in turn had multisystemic knock-on effects (in a ramifying cascade—RC), including widespread political instability. The ‘X’ symbol depicts compounding stresses (on the left-hand side) and crises (on the right-hand side). Adapted from: Homer-Dixon et al.^35^. Synchronous failure: the emerging causal architecture of global crisis. Ecology and Society, 20(3). https://www.ecologyandsociety.org/vol20/iss3/art6/^35^—licensed under CC BY 4.0.

As well as the stark implications for food insecurity of this entangled food-energy crisis, which have been the focus of the majority of the analysis on its human welfare implications, the oil price surge itself is estimated to have had a considerable impact on economic growth in many economies including in the US^42^ and Europe^43^. Figure 1 highlights, using Homer-Dixon et al.’s^35^ ‘synchronous failure’ schema, the entangled forces linking food and energy crises during this period.

The more recent 2022 global food crisis was linked intricately with an energy supply disruption and consequent price surge, following Russia’s invasion of Ukraine—an event that ‘triggered’ both energy and food crises^9^. But these have not evolved as crises in parallel; rather, they have become highly intertwined. Direct energy costs into agriculture and indirect costs through energy inputs into pesticides and fertiliser, can account for 40–50% of variable cropping costs in advanced economies^44^. Surging energy prices helped drive, and compounded with, surging food crop prices following the invasion, themselves already increasing following the post-COVID-19 lockdown economic recovery, which stretched supply chains, as well as extreme weather events affecting multiple regions, including floods in Pakistan and droughts in the Horn of Africa^45^. These triggers added to a number of long-term stressors, including a high degree of variability in cereal production over the past two decades, vulnerability to extreme weather, and relatively low yields related to limited access to credit and finance and regulatory uncertainties^46^. The consequences, with very high import dependence on Ukraine and Russian grains across the world, were devastating, with over 60 million more people in food crisis in 2022 compared to 2021^45^.

**Fig. 2:**
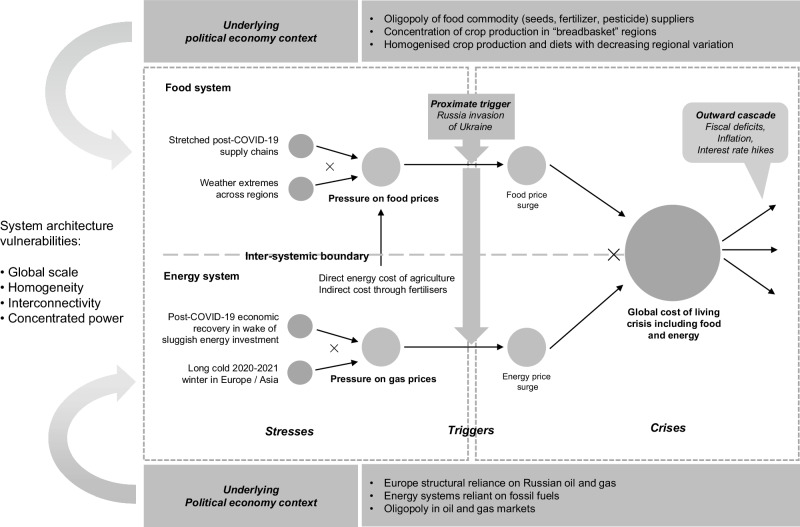
The 2022 global food and energy crisis. This figure is based on, but further develops, the elements introduced in Fig. 1. As before, the left-hand side depicts long-term stresses. Note that these include specific post-COVID-19 recovery-related stresses additional to those depicted in Fig. 1. In particular it should be noted that the ‘rising energy demand’ element of Fig. 1 was exacerbated by a sharp increase in demand in the post-COVID-19 economic recovery, as depicted in this figure. Similarly, for the global food system, the post-COVID-19 recovery stretched supply chains, adding to the stresses on the food system from rising demand, diminishing land availability and diminishing marginal returns to intensification as depicted in Fig. 1. Rather than a ‘Long-fuse big bang’ system overload as depicted in Fig. 1, this figure shows the trigger of the Russia invasion of Ukraine leading to the materialisation of both an energy and food crisis, stemming from these already stressed, interconnected systems. It also depicts new elements, specifically the underlying political economy context, including concentrations of political and economic power and reliance on a few providers of food and energy commodities, as well as other underlying systemic weaknesses. In food systems, these weaknesses include geographical concentration of production in breadbasket regions, as well as homogenised crop production. In energy systems, these weaknesses include reliance on relatively price-volatile fossil fuel resources. Together these political economy contexts give rise to what we term system architecture vulnerabilities, which make it more likely that intra- and inter-systemic risks and crises will compound and cascade. As with Fig. 1, the ‘X’ symbol depicts compounding stressors (on the left-hand side) and crises (on the right-hand side). Produced by authors.

A stylised schema, similar to that shown in Fig. 1, can be constructed for this food-energy crisis (Fig. 2). The schema adopts the new terminology of stresses being triggered into crises from Lawrence et al.^9^, but adds an underlying political economy context element to each system, to highlight the role of power, concentration and homogeneity in the system. Market power and lack of competition have been identified as instrumental in creating systemic risks in the run-up to the 2007–2009 Global Financial Crisis^47^ (and allowed huge profits to be made, for example by the world’s ‘big five’ global agro-commodity suppliers, during the 2022 crisis^48^), whilst homogeneity in financial portfolios similarly contributes to increased systemic risk^49^. More generally, high connectivity and homogeneity have been identified as important in potentially allowing tipping into cascading impacts across systems^35,50^.

These crises encompass key similarities. In both cases, they could be argued to be a ‘surprise’, given that the 2008 food price increases were not foreseen even a year earlier by major organisations including the IMF, World Bank and FAO^36^, whilst Russia’s invasion of Ukraine was not widely anticipated, even in late 2021^51^. Following the 2008 crisis, a number of initiatives were implemented to respond to the food system crises, including the Integrated Food Security Phase Classification (IPC)—to improve data collection and analysis—as well as a United Nations High Level Task Force on the Global Food Crisis—to coordinate responses^52^. The 2022 crisis saw the establishment of a Global Crisis Response Group on Food, Energy and Finance established by the United Nations in March 2022 and overseeing the Black Sea Grain Initiative to secure exports of grain and foodstuffs from Ukrainian ports^53^. The 2022 crisis saw far more humanitarian funding going towards food security compared to the 2008 crisis (some $12 billion in 2022^54^, compared to $3.5 billion in 2008^55^), but the 2022 figure funded only 61% of appeals^54^, compared to 92% in 2008^55^, indicating a severe funding shortfall given the scale of the crisis. It has been argued that lessons from 2008 crisis were not learned, with responses to that crisis, although establishing better data and monitoring, failing to build long-term resilience measures^52^ and thereby preventing the 2022 trigger forming a new crisis.

What, then, could have been done to systemically assess and respond to these crises? The two crises allow an identification of factors, and thus the key systems (including both within and outside food and energy systems) that must be considered in a more comprehensive analysis, that are important to account for in understanding the emergence and dynamics of these, as well as other, crises. Such a ‘retrodictive’ analysis is powerful in identifying factors driving actual crises—something which a speculative future crisis analysis might struggle to do, given the daunting list of potential factors and systems to assess. In terms of the 2008 crisis, these systems include: energy markets, to understand drivers of biofuel demand, oil supply and demand, price elasticities of energy demand; crop production systems, accounting for crop growth dependence on extreme heat, crop and biofuel production decisions, food stocks and buffers, international food commodity trade, fertiliser prices and their dependence on oil prices; the climate system and its response, in terms of temperature and precipitation extremes, to underlying warming; the role of economic and financial systems and their political economy; and policy and humanitarian response systems. Additional systems at the heart of the 2022 crisis include: geo-political systems and the risks of invasion and conflict; and economic systems and their recovery from shocks (in this case COVID-19).

Analysis of these crises can be used to derive a generalisable set of lessons for other risks and crises involving entangled systems. The analysis highlights the importance of several important steps, to understand their factors and dynamics systemically. First, an understanding of the system architectures themselves is required, including system stakeholders (those that hold market concentration and power; those vulnerable in them; those governing them) and the structure of trade and interconnectivity within them (as depicted in Fig. 2). In the cases outlined above, the ‘power’ holders include those placing embargoes on food and energy exports, those food and energy suppliers able to benefit from energy and food price rises, and financial speculators, whilst the vulnerable include the millions facing food insecurity and energy poverty.

In addition, the stressors, triggers and crises either in existence, or that have the potential to occur, are critical to try to identify ex ante. These are set out in Figs. 1 and 2, with: stressors stemming from falling productivity of agriculture and energy extraction, triggers stemming from extreme weather (linked to climate change) and geo-politics (Russia’s invasion of Ukraine), as well as disease (COVID-19’s stresses on supply chains); and crises cascading from financial speculation, as well as already-stressed energy and food markets. This will require diverse data and evidence, as well as cognitive diversity and thus a range of disciplines, including from economics, social sciences, history and—where for example interactions with climate and environment occur—the physical sciences.

The identification of responses, both effective and maladaptive, will be central to risk assessment, together with an identification of the synergies and trade-offs with other responses, such as the impact of grain export embargoes on food security in other countries, and the impact of gas import bans on electricity prices and fuel poverty. Finally, the long-term impact of responses, not just on risk preparedness, mitigation and adaptation, but on more fundamental transformation of systems away from inherently risky states and architectures, is essential if risk assessment is to be truly fit for purpose. There is evidence that some short-term responses, such as the Black Sea Grain initiative and diversification of natural gas imports from Russia, were effective in mitigating some of impacts of the 2022 crisis. But neither the 2008 nor 2022 crises were entirely exceptional; they were part of unequal food systems, unstable geopolitical landscapes and volatile energy markets. And as noted above, systemic resilience was not effectively built after the 2008 crises, leading to later vulnerabilities. Addressing systemic risk in the long-term needs to consider the deeper transformations required to make these systems inherently less risky—both to human societies and also to ecosystems, which suffered greatly through the extreme climate events, for example. Such a detailed and thorough set of considerations requires a series of analytical steps, to fully capture the factors and dynamics at play.

### The role of models and other analytical approaches to assess these crises

Much integrated analysis of climate, economy, agricultural and energy systems has been undertaken as part of energy planning and analysis of greenhouse gas emissions reduction pathways. This begs the question as to whether models are available and sufficient to understand crises such as those described in the previous section.

**Fig. 3:**
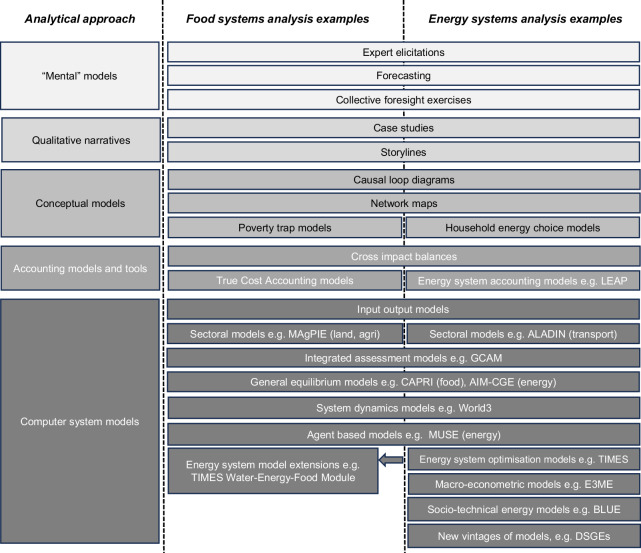
Catalogue of food and energy systems analysis approaches. This figure shows a range of analytical approaches and models, with specific examples given for food and energy systems, ranging from mental models which are not formalised into structured relationships between variables, to mathematical, computer system models which do represent such structured formalisation. Intermediate stages (qualitative narratives, conceptual models and accounting models and tools) represent an increasing extent of formalisation and mathematical structure. Grey-scale differentiation is added only to distinguish categories of analytical approach and has no inherent implication. Each of these analytical approaches can be incorporated into the systemic frameworks in Table 1. It should be noted that mental models can encompass very different boundaries of systems, depending on the question and scale of analysis. Full details in Supplementary Data 1. Produced by authors.

An inventory of differing approaches to analyse the operations of food and energy systems (Fig. 3), derived from a variety of literature-based sources, reveals similarities in approaches across both systems, along a taxonomy that ranges from purely qualitative to more quantitative approaches. In particular, there is a recognised distinction between mental models and mathematical models^56^ in food systems analysis, which is closely mirrored in energy systems analysis. The former entail a conceptualisation of food and energy systems and their operations, whilst the latter represent more formally structured sets of relationships between different elements of food and energy systems operations, codified into mathematical computer models. Additional approaches sit between purely mental models and mathematical models (qualitative narratives, and conceptual frameworks) to reflect an increasingly explicit formalisation and codification of features that underpin the operations of food and energy systems.

A detailed analysis of these different approaches (Supplementary Note 3) encompasses their key features, as well as an assessment of how they reflect particular food and energy systems operations, from a perspective of their ability to capture elements of intra- and inter-systemic risks and the crises that could result from them. These features include an assessment of their basic purpose, spatial granularity, geographic coverage, temporal granularity, food and energy system types, data input type, data demands, solution type (if relevant), links to other systems, representation (if any) of socio-political systems and power dynamics, and ability to capture non-linearities, shocks or tipping points, that are critical to systemic risks^57^.

This initial assessment suggests individual approaches could have gaps and limitations in their ability to assess both intra-systemic food and energy systems risks and inter-systemic risks between these and other systems. For example, more qualitative approaches on their own can potentially capture the detail and nuance of realities, including trans-contextual information^10^, that might be consequential to the emergence of crises resulting from the materialisation of risks, particularly if elaborated through detailed storylines and narratives. Such information might include knowledge of market concentration of commodity suppliers, vulnerability of communities relying on food, energy or other vital commodity imports, and the institutional or state capacity to respond to crises in different regions. However, these would lack quantitative power to compare across risks, support the evaluation or ranking of responses, or explore formally structured dynamics of crises, including potential speed and scale of spread. In addition, formal mathematical modelling can often reveal rapid and/or complex system dynamics (for example, exponential growth^58^ and network propagation) that might otherwise seem unintuitive or unlikely with qualitative methods alone.

On the other hand, more formal, codified and structured mathematical computer models may omit important factors driving risks and the contexts in which they emerge. In such cases, even where the models could be thoroughly initialised and calibrated with appropriate real-world data, they might still generate projections that deviate considerably from reality, if currently negligible drivers or processes grow to become dominant in the future^10^. This effect, known as the ‘Hawkmoth effect’, is a corollary to the better-known ‘Butterfly effect’, which reflects the deviation of model outcomes from real-world outcomes owing to initial data fed into the model deviating from reality. Both effects are of central importance to simulating systems exhibiting complicated (i.e. many-variable) and complex (i.e. emergent) features^59^. It should be noted that—just as with purely qualitative approaches - even mathematical, codified approaches rely on mental models for their construction. As such, this continuum from the purely qualitative to the more quantitative is not a dichotomy, but rather a gradient of the degree of codification of mental models.

Thus, although many aspects of the approaches considered above are highly applicable to the crises outlined, there is no singular approach that is suitable to comprehensively assessing and characterising the full, salient set of factors and dynamics at play in the crises described.

More critically, in many analytical approaches that have been used to examine crises, there is little assessment of long-term system goals, including questions around power, political economy and explicit considerations of risk and resilience for whom, from what, where and when. In addition, there are few truly integrated analytical approaches which encompass proximate factors from and to other systems, allowing endogenous dynamics, including feedback loops, to be examined. This is particularly true of equilibrium models, which do not allow long-run disequilibrium effects. In addition, a number of features of the historical crises examined suggest specific factors that should be taken into account but that rarely are, including a consideration of the role of responses in dampening or mitigating adverse impacts and feedback loops.

It should be noted that such post-hoc assessment of models that were not explicitly created for analysis of systemic risks and crises is arguably unfair—since models are designed to answer specific questions^59^, rather than any conceivable question that a user might decide to ask of them. Nevertheless, understanding potential gaps and shortcomings in analytical tools and approaches to address a given task (here systemic risk assessment) helps the undertaking of producing a detailed specification of what good practice to address that task should consist of.

## A methodological framework for systemic risk assessment

**Fig. 4:**
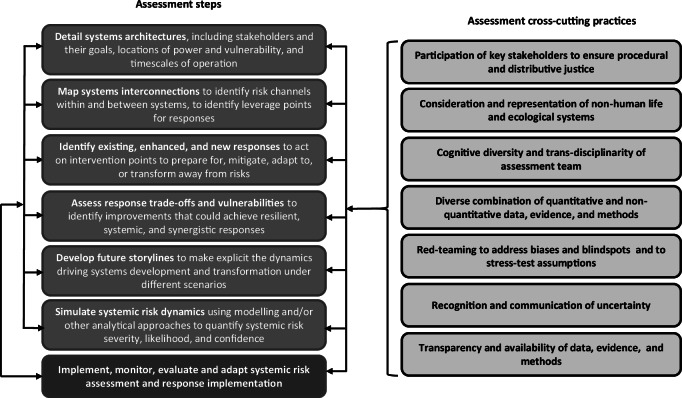
A generalisable systemic risk assessment methodological framework. The figure depicts (left-hand side) seven systemic risk assessment steps and (right-hand side) seven cross-cutting practices to be applied across each of the assessment steps. The steps are intended to be undertaken in the logical order shown, but the arrows indicate that that each step can and should if necessary, be returned to once other steps have been undertaken. As such, the framework is not prescriptive in its sequencing of steps. The arrow (left-hand side) from the ‘implement, monitor, evaluate and adapt’ step to the other steps is indicative that, once systemic risk assessments and responses have been implemented, they can and should, where necessary, lead to review and revision of each of the other steps. Produced by authors.

In light of the discussion on modelling limitations, rather than proposing an existing or new model or tool to address all of the steps necessary to undertake a systemic risk assessment, which could take years of development and empirical testing, calibration and verification, we instead propose a methodological framework (Fig. 4) to assess systemic risks that allows a combination of tools, models and analytical approaches. Utilising a variety of approaches to supplement, rather than replace, large yet still not comprehensive models, has been argued in climate and energy modelling, for example, given the urgency and complexity of decision-support needs in this area^60^.

Our methodological framework derives from an assessment of the salient features of the examined food-energy entangled crises, as reflected on and discussed during two intensive workshopping sessions (detailed in Supplementary Methods). These sessions identified each element of the framework as critical to fully understanding the observed food-energy crises which, via their deep interconnections to economic, political, environmental, social and technological systems, offers a generalisable set of steps to address polycrisis issues of entangled systemic risks within and across systems.

Specifically, although existing systemic risk assessment frameworks (as outlined in Table 1) acknowledge the need to explore the systems of concern to some extent, including the different system goals, our framework requires a more explicit consideration of power and political economy, deriving from the contextual factors identified from the recent historical food-energy crises (Fig. 2). This allows a deeper understanding of long term contextual factors that shape current systems, and the risks for different stakeholders (both human and ecological), which may be critical for avoiding undesirable resilience to unfavourable states for the most vulnerable.

In addition, virtually all existing systemic risk assessment frameworks include the practice, or potential, of mapping systemic interconnections, which should signal potential leverage points on which responses can act. However, our framework more explicitly considers fundamental transformations away from undesirable system states as part of its generation of potential response measures. This will require deep thinking and imagination on possible systems transformations, potentially far beyond the scope of specific policies or instruments aimed at risk mitigation. Such transformations include, for example, decarbonisation of energy at the same time as providing universal energy access and alleviating energy poverty, as well as building food system resilience and long-term food security, whilst restoring natural land and ecosystems. This is no simple task, but neither is it impossible. These transformations must happen at a systems, institutional, organisational, cultural and personal level.

Borrowing in particular from the Decision Making under Deep Uncertainty framework, our framework also considers explicit acknowledgment of response trade-offs and vulnerabilities, to push systemic risk assessors to find responses that will last the course, rather than requiring hasty re-configuring in light of unexpected shocks.

Many existing frameworks acknowledge the importance of storylines and narratives. Our framework explicitly calls for consideration of how risks could evolve to crises, compound and cascade within and between systems, both with and without response measures; and further deepening of these storylines, through modelling and other analytical approaches (such as those outlined in Fig. 3). In addition, and in common with most existing systemic risk frameworks, our framework includes the critical process of monitoring, learning and adapting to changing circumstances.

Finally, our proposed methodological framework, as with many other frameworks, includes cross-cutting ‘assessment practices’, derived from a broader conception of overarching principles around systemic risk assessment and response, specifically designed to address this global polycrisis^61^. These are clearly applicable to the food-energy crises examined earlier: ensuring participation of key stakeholders, including those most vulnerable to risks escalating into crises; centrality in assessment to non-human life and ecosystems, on which all social and economic activities depend (e.g. through providing food, fuel and fibre), as well as having intrinsic value in their own right; diversity in perspectives, to capture different factors and drivers of risks; diversity in data, evidence and methods, to ensure that the greatest number of these factors and drivers can be quantified and/or qualified; and active questioning and minimising of biases through stress-testing and red-teaming. Fit-for-purpose systemic risk assessment in the context of multi-faceted, ambiguous and fast-evolving interconnected risks must also actively communicate irreducible uncertainty, to ensure that assessment results are not seen as definitive or prescriptive where this is inappropriate. Finally, consideration of risk dynamics and responses will require detailed, transparent data, evidence and methods, capturing the value of both quantitative data sets and methods, as well as on-the-ground experience of people and communities facing risks and vulnerabilities. Organising this around a taxonomy of risks that measure elements of system architectures, long-term stresses, short-term triggers and resultant crises, as well as existing and potential responses, provides a logical method to catalogue and parse diverse risk-relevant data, evidence and methods (Supplementary Note 4).

A particularly distinct feature of our framework’s cross-cutting practices, compared to those of other frameworks, is around non-human and ecological system considerations. This recognises that systemic risk assessment, with deep roots in environmental degradation, cannot only be undertaken through an anthropocentric lens.

As with the other systemic risk frameworks, our framework offers a considerable departure from—and advance upon—traditional risk management as encompassed, for example, by the ISO 31000 standard^62^ (Table 2). This derives from: contextualisation of risks in terms of longer-term stressors, triggers and crises; a focus on inter-systemic connections and interactions; an explicit consideration of intervention points; analysis of risk cascades; and analysis of potential system transformations. In addition, the unique features of our framework, which make it particularly applicable to a polycrisis world, are its grounding in nature-centric practices and its explicit consideration of system architectures, in terms of the power, vulnerabilities and stakeholders that benefit or lose from current system operations and structures. This lends itself specifically to analysis of a polycrisis world, with deep drivers stemming from not just the Great Acceleration of socio-economic development since the mid-20th Century, but also the longer-term drivers of inequality, power and vulnerability that derive from longer-run processes of industrialisation, environmental destruction and extractivism, as well as colonialism. Any risk analysis that aims to delve deeply into the ways in which to respond to these elements of the polycrisis must reasonably contend with these issues.

**Table 2:** Comparison of key requirements for global systemic risk assessment across frameworks

	Systems architectures: Exploring system goals	Systems architectures: Power and vulnerability	Systems interconnections: Stress, trigger, crisis framing	Systems interconnections: Risk linkages and leverage points	Identification of responses: Existing, enhanced, new	Identification of responses: Exploration of transformations	Assessment of response trade-offs & vulnerabilities	Development of storylines	Simulation of risk dynamics	Implementation, monitoring, learning & adaptation	Principles & practices including nature-centricity
Cascade Institute Polycrisis Analysis Guide^16^	(Y)	(Y)	Y	Y	(Y)	(Y)	(Y)	Y	(Y)	(Y)	N
MYRIAD-EU 6-Step framework^17^	(Y)	(Y)	(Y)	Y	Y	(Y)	(Y)	Y	Y	Y	(Y)
Systems Thinking Toolkit for UK Civil Servants^18^	Y	(Y)	(Y)	Y	Y	(Y)	(Y)	Y	Y	Y	Y
SysRisk approach^19^	Y	(Y)	(Y)	Y	Y	N	Y	Y	(Y)	Y	N
IRGC Guidelines on Systemic Risk Governance^20^	Y	(Y)	(Y)	Y	Y	Y	Y	Y	Y	Y	(Y)
Infrastructure resilience stress-testing approach^21^	(Y)	(Y)	(Y)	Y	(Y)	(Y)	(Y)	(Y)	Y	(Y)	N
DMDU Methods^22^	(Y)	(Y)	(Y)	(Y)	Y	(Y)	Y	Y	Y	Y	(Y)
ISO Risk Management Guidelines (ISO 31000)^62^	(Y)	N	N	N	Y	N	N	Y	N	Y	(Y)
ASRA SRA methodological framework	Y	Y	Y	Y	Y	Y	Y	Y	Y	Y	Y

*Y*  Yes/fully, (*Y*)  Partially, *N*  No Note that the frameworks in this table are those in Table 1, with the addition of the ISO risk management framework 31000, to show how systemic risk management compares to standard risk management. ASRA denotes the Accelerator for Systemic Risk Assessment, the systemic risk assessment working group of which designed the framework discussed in this paper.

## Operationalising the framework

This methodological framework serves to avoid major omissions in assessing systemic risks. The different steps in the framework are not necessarily prescriptive in their sequencing. For example, the generation of potential responses to address intra- and inter-systemic risk cascades and effect a transformation to lower-risk, more resilient systems, is an activity that could be performed before, in parallel with, or after, assessment of risks within scenario exercises. In addition, agreeing on future system states as part of scenario design may be a more tractable first step than identifying all risks, system boundaries and interconnections. Depending on the objectives of the analysis, it could be that such scenarios are aimed at assessing a ‘no response’ set of potential futures, to outline a counterfactual of how systemic risks could compound and cascade and result in impacts without intervention, to justify responses. Or it could be that the scenarios are designed with incorporation of responses from the outset, to identify the most ‘robust’ responses i.e. those which perform best, in terms of addressing risks, in a variety of different scenarios (as part of a robust decision-making process^63^).

Applying the framework to the crises described above demonstrates that it could capture a large range of the consequential forces and dynamics of those crises (Table 3). However, a fully comprehensive undertaking of all these steps could take several weeks to months (given the required timescales of comparable futures visioning and planning exercises), and—if it requires the development of new computer system models or tools—considerably longer. As such, as a minimum our methodological framework should be applied as a ‘checklist’ to understand what, if any, additional tasks could and should be undertaken with greater budget, time or other resource.

**Table 3:** Applying the methodological framework to the historical food-energy crises

	Key questions to consider	Examples of how food-energy crises addressed
Detail systems architectures	• What are the goals of the system(s)? • Who do these systems serve? • What systems does it/do they closely connect to? • Why are we concerned about harms from these systems?	Asking these questions would identify critical contextual factors of food and energy systems: • The power and profits of large energy and agro-commodity companies with control of energy and food supply and trade; • The vulnerability of people in energy and food commodity-importing countries to supply shocks and price rises; • The systems to which food and energy systems are closely connected and that lead to reasons for concern, including: food system dependence on energy prices through fertiliser and agriculture machinery costs; both systems’ influence on socio-economic systems in different countries whose revenues depend on exports of these products; ecosystem and climate system implications from pollutants from these systems.
Map systems interconnections	• What is the best way (given available time and resources) to map interconnections between systems? • What more could/should be done to capture interconnections in more detail?	Approach would identify: • Inter-systemic links e.g. using participatory systems mapping to identify major causal drivers of food-energy crises (Supplementary Fig. 3); • Cascading consequences to the economy, social, financial, environmental systems from crises in food and energy systems, including possible behaviours like the role of financial speculation, food and gas export embargoes.
Identify existing, enhanced and new responses	• What are the critical leverage points of the system(s)? • What are the existing response types for the system(s) of focus? • What are potential enhanced and new response types? • How could the system(s) transform more fundamentally and what would be required?	Participatory processes would utilise: • Databases of existing response types and design of new responses to allow identification of suitable, systemic responses(Supplementary Note 3). These could include: diversification away from gas towards renewables and energy efficiency; larger local grain stores to mitigate local food price volatility; lower-carbon intensity energy use to respond to climate heating; multilateral agreements to provide emergency energy and food supplies during times of crises; • Foresight and scenario exercises to imagine transformative changes towards less risky food and energy systems, considering their interconnections with other systems and the potential risks facing them.
Assess response trade-offs and vulnerabilities	• What are the major trade-offs of envisaged responses? • Where are vulnerabilities in the responses, in light of future possibilities? • What are the critical leverage points of the system(s)?	Analysis would use systems mapping, response generation, identification of systems goals and power to identify: • Response trade-offs e.g. securing of alternative gas supplies to lock-in to new LNG port infrastructure, thereby lengthening dependence on carbon-intensive gas; • Response weaknesses, e.g. lack of resilience of some agricultural responses in light of climate extremes.
Develop future storylines	• How could risks propagate/cascade/compound/subside, considering response/no response storylines? • What are the implications of these scenarios across a variety of system metrics/measures?	More explicit use of scenarios could highlight: • Urgency of response options and resilience measures, including specific regional dependence on fossil fuels with volatile prices; • Utility of investment in low-carbon, regenerative, local agriculture to protect against international food price shocks; • Impact of multilateral agreements to provide emergency energy and grain supplies.
Simulate systemic risk dynamics	• Can storylines be simulated analytically e.g. in models? • If applicable, what are the size of the adverse impacts/avoided impacts? • With what likelihood and confidence levels?	If available and applicable, appropriately calibrated models including integrated assessment, shock propagation, system dynamics and agent-based models could be used to explore: • The impacts and avoided impacts of response measures under different scenarios; • Changes in risk likelihoods and/or frequencies of occurrence as a result of different responses (if run in stochastic modes).
Implement, monitor, evaluate, adapt	• How are risk and risk cascade dynamics developing? • How effective are responses? • What adaptations, revisions and course corrections are required?	Detailed risk monitoring measures for food and energy systems would collect evidence (and identify evidence gaps) including around: • Import dependence, indigenous resources and diversity of resources around food and energy supply; • Local resilience to crises through state capacity, transport networks, social capital and other responses; • Procedures to respond with enhanced and new measures if/when risks reach pre-defined thresholds.

This methodological framework is intended to be applied in a number of different contexts and by different stakeholders, drawing on its modularity and flexibility. These are intended to include: governments undertaking national risk assessments; research and educational institutions, to better understand the systemic risks stemming from, for example, climate change and biodiversity loss, including tipping points and non-linearities; financial institutions, as part of capital adequacy and liquidity stress tests in the face of multiple, cascading risks as seen during the Global Financial Crisis of 2007–2009, and COVID-19 pandemic of 2020 onwards; and civil society groups, understanding risk interactions and responses for vulnerable communities, households and citizens at local levels.

There is no shortage of critical nexus issues which could form the context of this methodological framework, including extending energy-food nexus analysis to encompass water, security, social justice and climate more explicitly^64^, as well as to new technologies such as artificial intelligence and their risk interactions with other systems. Table 4 serves to highlight the application of this framework to an example nexus issue, weaving together some of the highest-ranked global risks (in terms of severity)^65^.

**Table 4:** Example application to a plausible future systemic crisis deriving from foreseen risks

Example systemic risk issue in a polycrisis world	Use of framework to analyse its dynamics, through example questions to explore at each step
Incursion of AI into energy sector in the context of a warming climate	**Detail systems architectures:** Who controls AI technologies and what are their incentives? Who is vulnerable, through biases, lack of access, job losses? What are governments’ incentives to support and regulate these technologies? What forces are driving the energy sector towards net-zero? How just are these transitions, to both human societies and ecosystems and who is in control of low-carbon technologies and measures? **Map systems interconnections:** How will AI drive/hinder net-zero transitions, through e.g. increased low-carbon technology innovation, increased energy demand, more effective fossil fuel exploration/extraction? What will AI’s impact on mis/disinformation and geo-political cooperation be and how will this affect global climate action? How will a warming climate affect energy technology deployment, e.g. as a result of climate impacts on energy infrastructure? How will low-carbon measures affect ecosystems? **Identify existing, enhanced and new responses:** What policies, treaties, regulations, plans and strategies have been, or could be, effective at driving low-carbon transitions, promoting and enforcing responsible and equitable AI, protecting biodiversity and land systems and infrastructures? **Assess response trade-offs and vulnerabilities:** Where are the synergies and trade-offs, both across human societies and ecosystems and how can these be addressed/enhanced respectively? What future disruptions could responses be vulnerable to, for example through social, private sector, or political backlash, or to a deterioration in international cooperation? **Develop future storylines:** How would the above responses intervene to prevent, adapt to, mitigate and transform away from the potential risks identified? What further implications are there for societies and ecosystems along these pathways, both with responsible AI, utilised to accelerate a nature-positive low-carbon transition, and with unregulated AI, destabilising the transition and causing harms to societies and ecosystems? **Simulate systemic risk dynamics:** How, if at all, can computer systems models and other analytical approaches be used to quantify risk likelihoods and severities in the above scenarios? For example, through combining integrated assessment models of energy, land, climate and economic development with expert elicitations of the implications of AI. What is our confidence level in these quantifications? **Implement, monitor, evaluate, adapt:** What changes and course corrections are needed along the net-zero transition? In light of evolving power and societal dynamics around AI usage, what new/adjusted risk analysis is required, as new risk interconnections emerge and what does this imply for adjustments and revisions to the above steps?

A full application of underlying research and stakeholder engagements, as well as storylining, model parametrisation and implementation, could in theory take many months, or even years. But modules of the framework, particularly around identification of system objectives, interconnections and risk transmission channels, as well as around exploration of responses at key intervention points, could be undertaken in participatory workshops. Indeed this framework is currently being applied to a number of different contexts, scales and geographies, to fine-tune and adapt its applicability according to available skills, data and evidence, models and tools and according to different forms and extents of stakeholder engagement. In many cases these pilots, spanning analysis of interacting risks for slum-dwellers in São Paulo to implementation of the global biodiversity framework in a range of countries^66^ have focused on these workshop-based approaches, with new insights on risk interconnections and response intervention points being successfully identified.

One pilot, for example, focused on analysis of interacting risks to those involved in food production through farming and fishing in the lower, middle and upper zones of the Volta River Basin in Ghana, has concentrated on risks to and from food systems in this basin. Such systems have experienced growing stresses, triggered into resulting crises, both to and from local environmental pollution (for example from leaching of fertilisers from land into the waterways), as well as losses to maize crops due to extreme droughts, storm floods and dam spillages. These risks and resultant crises have been exacerbated by food price volatility, high levels of debt, as well as a high dependence on supplies of agricultural machinery from overseas. Multi-stakeholder workshops involving government officials, food producers, media experts, retailers and non-governmental organisations have developed detailed systems maps linking these factors. Furthermore, the pilot has identified the (often limited) effectiveness of existing government responses focused on individual symptoms, rather than underlying systemic risk causes, of crises. Such responses, including encouragement to plant drought-resistant crop varieties, better training and education for farmers and subsidies for farming inputs such as seeds, have not proven adequate to the scale and interconnectedness of the risks. This research has also highlighted the lack of adequate data and evidence to quantify and characterise the drivers and incidences of these risks and in particular their interactions. It has therefore signalled the need for a more holistic assessment of risks and their interconnections, as well as the trade-offs and vulnerabilities of risk responses, in order to transform the often-devastated farming and fishing systems of this river basin to a more sustainable and resilient state.

Indeed, all pilots have signalled the need for greater data and evidence gathering, to fill important gaps, for example on early warning systems and risk data and evidence and tools, supporting other evidence around this need^67^. Further application of the framework is intended to help highlight the skills, capabilities, institutional commitment and structures required to support systemic risk assessment. Such capacities to undertake enhanced systemic risk thinking are critical around both assessment and the design and implementation of responses. Guidance directing such systemic risk assessments (Supplementary Note 2) as well as information on available data and evidence to aid the process (Supplementary Note 4) will be reviewed and refined in light of lessons learned.

## Discussion

Our systemic risk assessment methodological framework integrates and builds on existing risk assessment practices to specifically address the interconnected nature of current systemic risks and crises. A number of advances are still required, however, in order that thorough systemic risk assessments can be undertaken on the basis of this framework.

First, the challenge of engaging a range of participants in risk assessment remains. Structural biases favour those that can engage in person through ease of travel, or online through adequate internet access and technological literacy. This could easily exclude communities with valuable local knowledge but little engagement in mainstream participatory processes. To remedy this, intentional steps are required to make stakeholder processes as inclusive as possible, providing not just a ‘place at the table’ but also a process that meaningfully attends to power imbalances between marginalised voices and incumbents^68^. Second, advances must be made in the field of modelling and analysis to provide the kind of characterisation and potentially the quantification of risks (likelihood, severity and confidence) that will be necessary to equip budget-conscious actors (be they governments, businesses, civil society or financial institutions) with appropriate decision-support. Such analytical methods must incorporate a representation of complexity science, resilience theory and network theory to be fit for purpose in the context of systemic risk^69^. Several recent advances have been made in modelling, including in particular agent-based models, which have the potential to simulate the emergence of macro-level system dynamics from micro-level agent interaction, making them potentially suitable to the analysis of systemic risk dynamics^70^. Third, key steps in this framework, including around the consideration of enhanced and new risk responses, as well as future storylines including systems transformations, will require a high degree of imagination and futures literacy. This is an under-represented skillset across institutions and organisations, owing to barriers including lack of organisational culture and lack of experience^71^. Fourth, the framework’s drive towards nature-inclusivity and nature-centricity is unlikely to be rapidly adopted by many actors, that still either see nature as separate from humanity, or that measure and treat the value of nature as instrumental to society and the economy, rather than intrinsically valuable in its own right. A much more integrated treatment of nature in systemic risk assessment is essential, recognising that societies and economies are not only dependent on, but an integral part of, the natural environment^72^.

The framework is intended to stimulate reflection and discussion on each of these challenges, though it cannot address them on its own. To do so will require widespread application of the framework through further participatory piloting, combined with model and analytical tool development, as well as a broader development of skills such as futures thinking.

These challenges notwithstanding, the framework’s modularity, highlighting the necessity to think deeply about systems, their architecture, objectives and stakeholders, as well as systems interconnections, makes it a tractable way of considering elements that are often overlooked (or implicitly assumed) in more detailed modelling and analytical exercises. Utilising this framework even as a checklist to ensure critical stakeholders and elements of analysis are not omitted would be valuable, even if the time and resources required for a full application of all steps is unavailable.

In summary, better and more holistically understanding the systemic risks that are driving the global polycrisis and designing appropriate responses to those risks is long overdue. A standardised—yet modular and flexible - procedure for doing so could be critical in preparing for, adapting to and mitigating the next global systemic crises, and transforming to a future where such crises are less likely and less severe.

## Supplementary information

Supplementary information

Description of Addtional Supplementary Files

Supplementary Dataset 1
