# Supplementary material for: A systemic risk assessment methodological framework for the global polycrisis
Source: Nat Commun. 2025 Aug 14;16:7382. doi: 10.1038/s41467-025-62029-w (PMC12354749; doi:10.1038/s41467-025-62029-w)
Supplement: Supplementary file 1 — Supplementary information [file 41467_2025_62029_MOESM1_ESM.pdf]

Supplementary Information for:

A systemic risk assessment methodological framework for the global polycrisis

Ajay Gambhir[1, 2], Michael J. Albert[3], Sylvanus S.P. Doe[4], Jonathan F. Donges[5], Nadim Farajalla[6], Leandro L. Giatti[7], Haripriya Gundimeda[8], Sarah Hendel-Blackford[1], Thomas Homer-Dixon[9], Daniel Hoyer[10, 11], Sumaya Adan[12], David Jacome-Polit[13, 14], Luke Kemp[15], David Korowicz[16], Zora Kovacic[17], Jan Kwakkel[18], Laurie Laybourn[19], Robert Lempert[20], Ayan Mahamoud[1], Tom H. Oliver[22], Ivana E. Pavkova[22], Joseph Ponnoly[23], Vishwas Satgar[24], Megan Shipman[9], Jana Sillmann[25, 26], Nick Silver[27], Samuel Stevenson[1], Ruth Richardson[1].

[1] Accelerator for Systemic Risk Assessment (ASRA), Washington, DC, USA
[2] Grantham Institute, Imperial College London, London, UK
[3] School of Social and Political Science, University of Edinburgh, Edinburgh, UK
[4] GeoSustainability Consulting, Adabraka-Accra, Ghana
[5] Earth Resilience Science Unit, Potsdam Institute for Climate Impact Research, Member of the Leibniz Association, Potsdam, Germany
[6] Lebanese American University, Chouran, Beirut, Lebanon
[7] School of Public Health, University of São Paulo, São Paulo, Brazil
[8] Department of Economics, Indian Institute of Technology Bombay, Mumbai, Maharashtra, India
[9] Cascade Institute, Royal Roads University, Victoria, BC, Canada
[10] Complexity Science Hub. Vienna, Vienna, Austria
[11] Societal Dynamics (SoDy), Toronto, Canada
[12] Oxford Martin School, University of Oxford, Oxford, UK
[13] ICLEI, Bonn, Germany
[14] TU Delft, Delft, Netherlands
[15] Centre for the Study of Existential Risk, Cambridge, UK
[16] Korowicz Human Systems, Balcarrick, Donabate, Co. Dublin, Ireland
[17] Urban Transformation and Global Change (TURBA) Lab, Universitat Oberta de Catalunya, Barcelona, Spain
[18] Faculty of Technology, Policy and Management, TU Delft, Delft, Netherlands
[19] Global Systems Institute, University of Exeter, Exeter, UK
[20] Frederick S. Pardee Center for Longer Range Global Policy and the Future Human Condition, RAND Corporation, Santa Monica, USA
[21] School of Biological Sciences, University of Reading, Reading, UK
[22] TMP Climate, Tokyo, Japan
[23] Cinfodens Consulting, Pearland, TX, USA
[24] School of Social Sciences, University of Witwatersrand, Wits, Johannesburg, South Africa
[25] Center for International Climate Research, Oslo, Norway
[26] Research Unit Sustainability and Climate Risks, University of Hamburg, Hamburg, Germany
[27] Bayes Business School, London, UK

Supplementary Items List:

Supplementary Note 1. Key terms and concepts in systemic risk and polycrisis.................................... 3
Supplementary Note 2. Draft guidance to support the ASRA SRA methodological framework 4
Supplementary Note 3. Detailed analysis of food and energy system tools and approaches 13
Supplementary Note 4. Data sources for supporting systemic risk analysis.. 13
Supplementary Methods. Approach to determine SRA framework 15
Supplementary references 19

Supplementary Note 1. Key terms and concepts in systemic risk and polycrisis

There are many different terms around the concepts discussed in the main manuscript[1-5], specifically surrounding how the complex adaptive socio-economic and environmental systems in which we operate are vulnerable to, and in fact generate, risks with the potential to spread and affect whole systems.

Supplementary Table 1: Key concepts underlying polycrisis and systemic risk

Key concept	Definition	Reference
Complex Adaptive Systems	Systems in which large networks of components with no central control and simple rules of operation give rise to complex (emergent and non-trivial) collective behaviour, sophisticated information processing, self organisation, and adaptation via evolution and learning.	Mitchell (2009, p13)[1]
Multi-hazard Risk	Risk generated from multiple hazards and the interrelationships between these hazards (but not considering interrelationships on the vulnerability level).	Zschau (2017)[2]
System of Systems	A system that involves several systems "that are operated independently but have to share the same space and somehow cooperate".	IEEE (2014)[3]
Systemic Risk	The threat that individual failures, accidents, or disruptions present to the system through the process of contagion.	Centeno et al. (2015)[4]
Polycrisis	The causal entanglement of crises in multiple global systems in ways that significantly degrade humanity's prospects.	Lawrence et al. (2024)[5]

Supplementary Note 2. Draft guidance to support the ASRA SRA methodological framework

This section describes the guidance which is intended to accompany ASRA's systemic risk assessment (SRA) methodological framework and the associated cross-cutting practices to utilise throughout each step. It is intended as training and guidance material for any organisations or communities undertaking systemic risk assessments. The framework is set out in Supplementary Fig. 2 below.

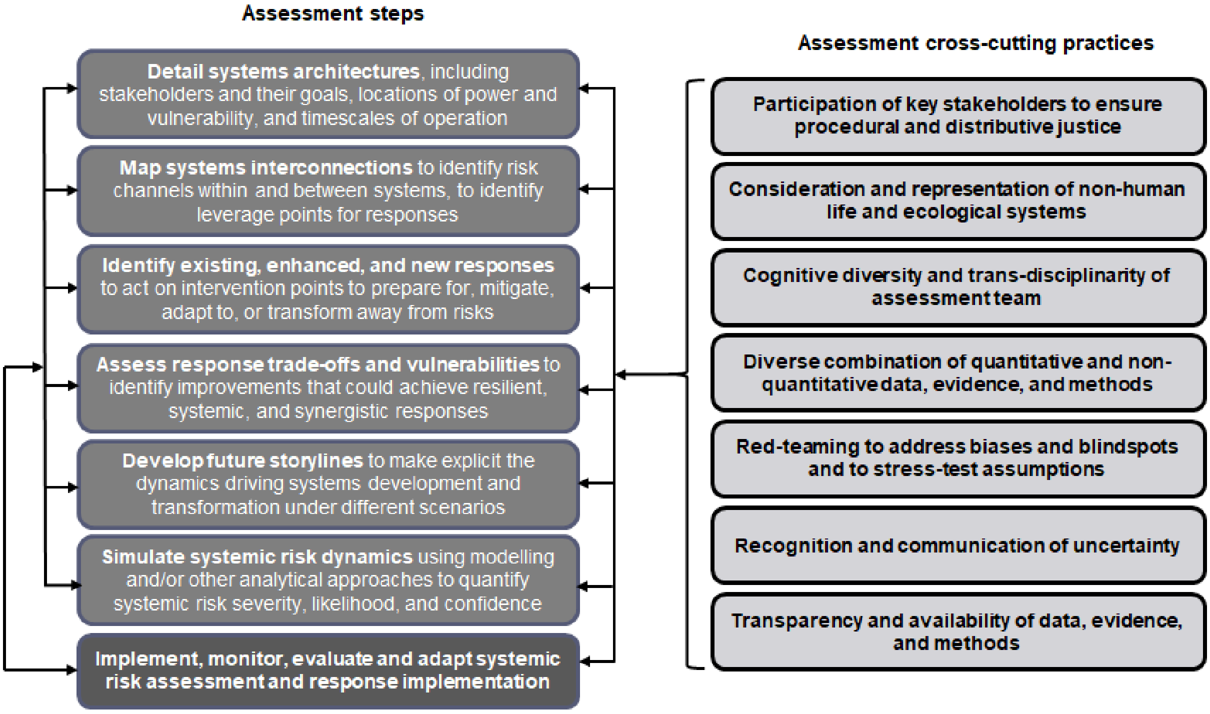

Supplementary Figure 1: ASRA's Systemic Risk Assessment methodological framework. *The figure depicts (left-hand side) seven systemic risk assessment steps and (right-hand side) seven cross-cutting practices to be applied across each of the assessment steps. The steps are intended to be undertaken in the logical order shown, but the arrows indicate that that each step can, and should if necessary, be returned to once other steps have been undertaken. As such, the framework is not prescriptive in its sequencing of steps. The arrow (left-hand side) from the "implement, monitor, evaluate and adapt" step to the other steps is indicative that, once systemic risk assessments and responses have been implemented, they can and should, where necessary, lead to review and revision of each of the other steps. Produced by authors.*

It should be noted at the outset that the framework is not prescriptive in terms of the analytical tools, resources, available stakeholders and perspectives and numbers of scenarios or futures explored. It is, rather, a set of guidelines to steer discussions and analysis on systemic risks and their interactions, so as to enhance the likelihood that the most important risk emergence and propagation dynamics, data, evidence and perspectives are captured and characterised. The more thoroughly each step is undertaken, the more complete and holistic such assessment is likely to be. The following sections describe the envisaged approach to each of the above steps in further detail.

Detail systems architectures

A large number of approaches, tools and models can be used to identify and estimate systemic risks both within systems and between them. At the heart of all of them are mental models and thought processes, encompassing imagination of what could be a risk, how it could materialise into a crisis, and how risks and crises could propagate through systems and beyond.

As such, any operationalisation of the SRA methodological framework specified here should undertake an initial exploratory exercise that tackles key questions. First, it should consider what is the system or set of systems of particular concern, including their geographical scope, their scale(s), the time period over which risks may arise and turn to crises, and the time period over which those risks and crises could propagate. It should then consider what these systems provide, and for whom, who holds the power within them, who is potentially vulnerable within them and from what risks, and what is at stake if risks materialise into crises. It should also identify the systems to which those systems of central focus connect, and how they do so, in terms of possibilities of risks and crises from those systems affecting the systems of focus, and vice versa. A critical question to consider is the reasons for worrying about the risks within these systems now and in a specified future time period, in terms of both the severity and likelihood of the risks and whether - and if so how - these risks could scale and escalate, or replicate, from local to regional and from regional to global scales.

These questions, and an attempt to address them, form the essence of the systemic risk assessment. One defining feature of these questions is around system goals and in particular an overt focus on the stakeholders in the system. This brings explicit consideration of system architecture into play, including where power lies in the different elements of the system, and thus who is at risk. As a result, this initial systems scoping should be done in a participatory manner, bringing in views, evidence and perspectives from all relevant stakeholders.

Map systems interconnections

The stress-trigger-crisis framing (derived from Ref[6]) utilised in the data and evidence gathering step could also be used to – as a related step to gathering data and evidence - map interlinkages between systems. It should be noted that there is a large range of potential methodologies for systems mapping. There are many different techniques as illustrated in Supplementary Fig. 2, ranging from qualitative to quantitative methods, and with foci ranging from specific interventions to system levels[7].

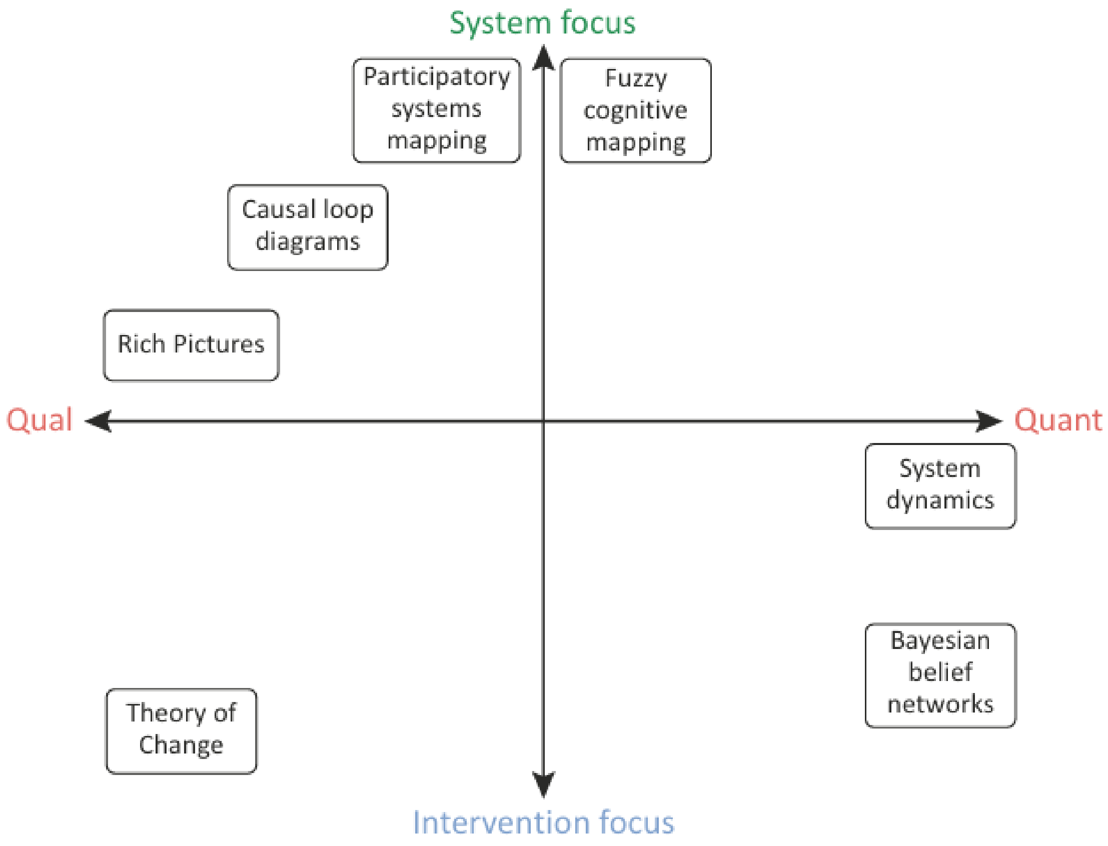

Supplementary Figure 2: Taxonomy of systems mapping techniques. *The figure shows where different systems mapping approaches sit along two axes, ranging from (horizontal axis) qualitative on the left-hand side, to quantitative on the right-hand side, and (vertical axis) approaches with a focus on specific interventions at the bottom, to approaches with a focus on whole systems at the top. Reproduced from: Barbrook-Johnson, P., & Penn, A. S. (2022). Introduction. In Systems Mapping: How to build and use causal models of systems (pp. 1–19). Springer. https://doi.org/10.1007/978-3-031-01919-7_1 [7] — licensed under CC BY 4.0.*

But in practice any structured approach to specifying more fully the system characteristics, dynamics, connections and interactions that create risks and crises is a useful and appropriate way of building on those questions asked in the first step. Supplementary Figure 3 shows a stylized set of causal links to and from regional food price rises, based on a combination of dynamics experienced during the 2008 and 2022 global food-energy crises. It should be noted that this is not a comprehensive set of links but a stylised diagram illustrative of many proximate and related causes of regional food price increases as experienced in past crises.

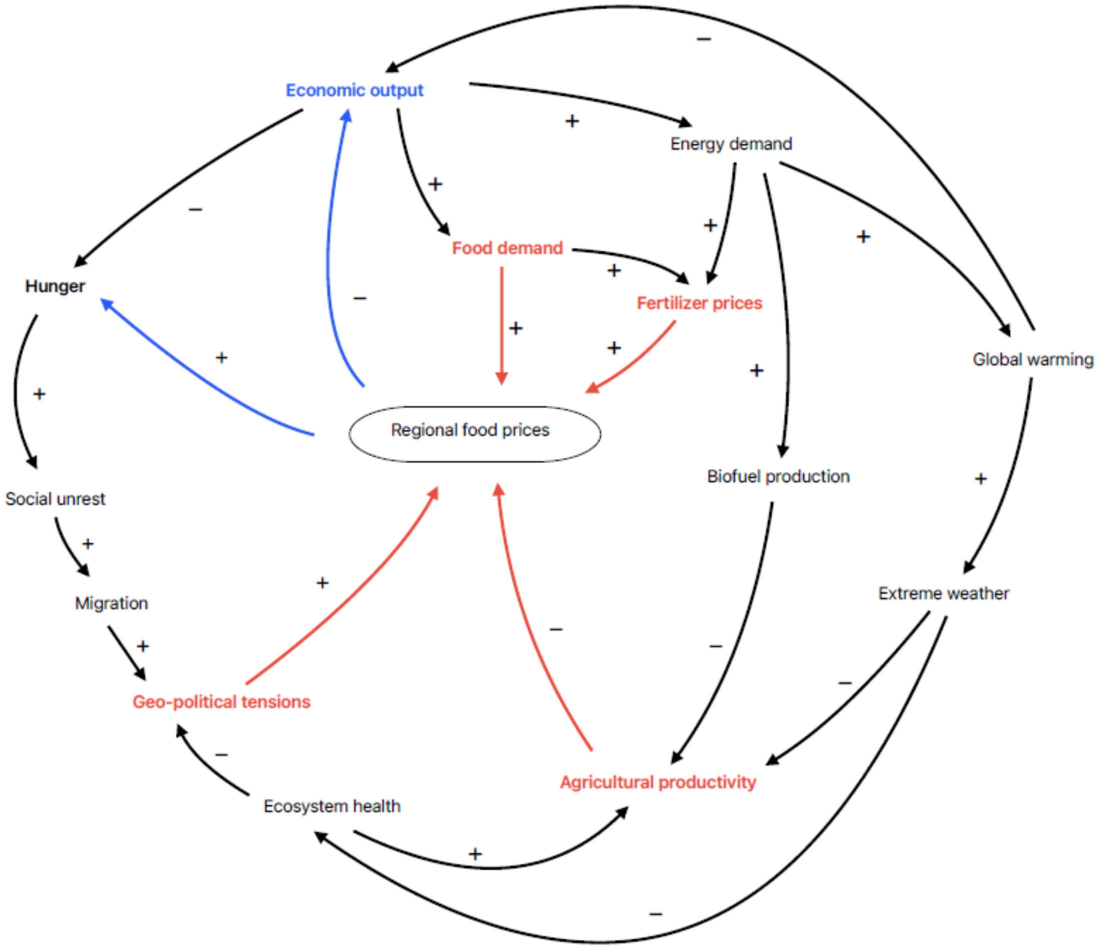

Supplementary Figure 3: Positive (+) and negative (-) causal links*. Authors' own figure, based on detailed analysis of 2008 and 2022 global food-energy crises). A "+" label indicates that where the casual variable increases (decreases), this leads to an increase (decrease) in the dependent variable. A "-" label indicates that an increase (decrease) in the causal variable leads to a decrease (increase) in the dependent variable. Red labels and arrows capture direct drivers of food price changes, whereas blue labels and arrows capture direct consequences.*

Identify existing, new and enhanced responses

As illustrated in Supplementary Fig. 3, the consideration of feedbacks between systems includes a need to account for both reinforcing and dampening feedbacks – in other words, those feedback loops that can make initial crises turn into even greater ones, or those feedback loops that can reduce crisis levels. Inherent in this latter case will be a consideration of possible responses to the systemic risks faced and the intervention, or leverage, points on which those responses should act. A full systemic risk assessment should consider the different responses to the risks, in terms of those that can either help to mitigate, prepare for, or adapt to risks, or even fundamentally transform systems to a less risky state. The generation of potential responses is thus important in considering the ways in which risks might be prevented from turning into crises, or how crisis cascades could be avoided or mitigated.

Response generation and assessment should also consider particular intervention, or leverage, points within and between systems, such that these points, be they temporal or spatial, can be identified. Supplementary Figure 4 shows Donella Meadows' famous example of different leverage points to intervene within a system[8], in order to achieve systems change. Whilst not the only structured

framework for thinking through different leverage point possibilities, it is nevertheless a fairly comprehensive and thus useful guide to help explore possible responses.

12. Constants, parameters, numbers (such as subsidies, taxes, standards)
11. The sizes of buffers and other stabilizing stocks, relative to their flows
10. The structure of material stocks and flows (e.g. transport networks, population age structures)
9. The lengths of delays, relative to the rate of systems change
8. The strength of negative feedback loops, relative to the impacts they are trying to correct against
7. The gain around driving positive feedback loops
6. The structure of information flows (who does and does not have access to information)
5. The rules of the system (such as incentives, punishments, constraints)
4. The power to add, change, evolve, or self-organize system structure
3. The goals of the system
2. The mindset or paradigm out of which the system – its goals, structure, rules, delays, parameters - arises
1. The power to transcend paradigms

Supplementary Figure 4: Meadows' 12 intervention points to achieve systems change. *This figure shows the different ways in which systems can be changed, in increasing order of effectiveness (where 1 is the most effective). Produced by authors, using information from Donella Meadows' essay "Leverage Points: Places to Intervene in a System"* [8].

In developing response measures and strategies, the ASRA Systemic Risk Response (SRR) criteria[9] should be referred to and considered, to identify responses that are more likely to be fit-for-purpose in a systemic risk context.

Assess response trade-offs and vulnerabilities

The response generation step is by no means likely to yield trade-off-free responses, since responses to deal with one risk may create a new risk. This is why a consideration of trade-offs should be undertaken, to understand the potential advantages and disadvantages, winners and losers, and the compensation or other mechanisms available.

This step should also encompass an assessment of vulnerabilities of responses to future possibilities, drawing from approaches such as Decision-Making under Deep Uncertainty (DMDU) and Robust Decision Making (RDM) which allow a testing of the vulnerability or robustness of different strategies under a range of future conditions[10].

Develop future storylines

Once the systems' goals, architecture and risk and crises interconnections, as well as responses and their leverage points, have been considered and mapped, the discussion of potential future scenarios – both with and without responses - can be more fully considered. Storyline creation consists of developing a narrative on how the systems of focus and risks surrounding them could occur and develop in the future. Such narratives should ideally achieve a degree of internal consistency and real-world feasibility.

Supplementary Figure 5 shows an example from the Network for Greening the Financial Services (NGFS) [11] which sets out both "transition" risks to reducing greenhouse gas emissions, as well as physical climate risks, in four quadrants.

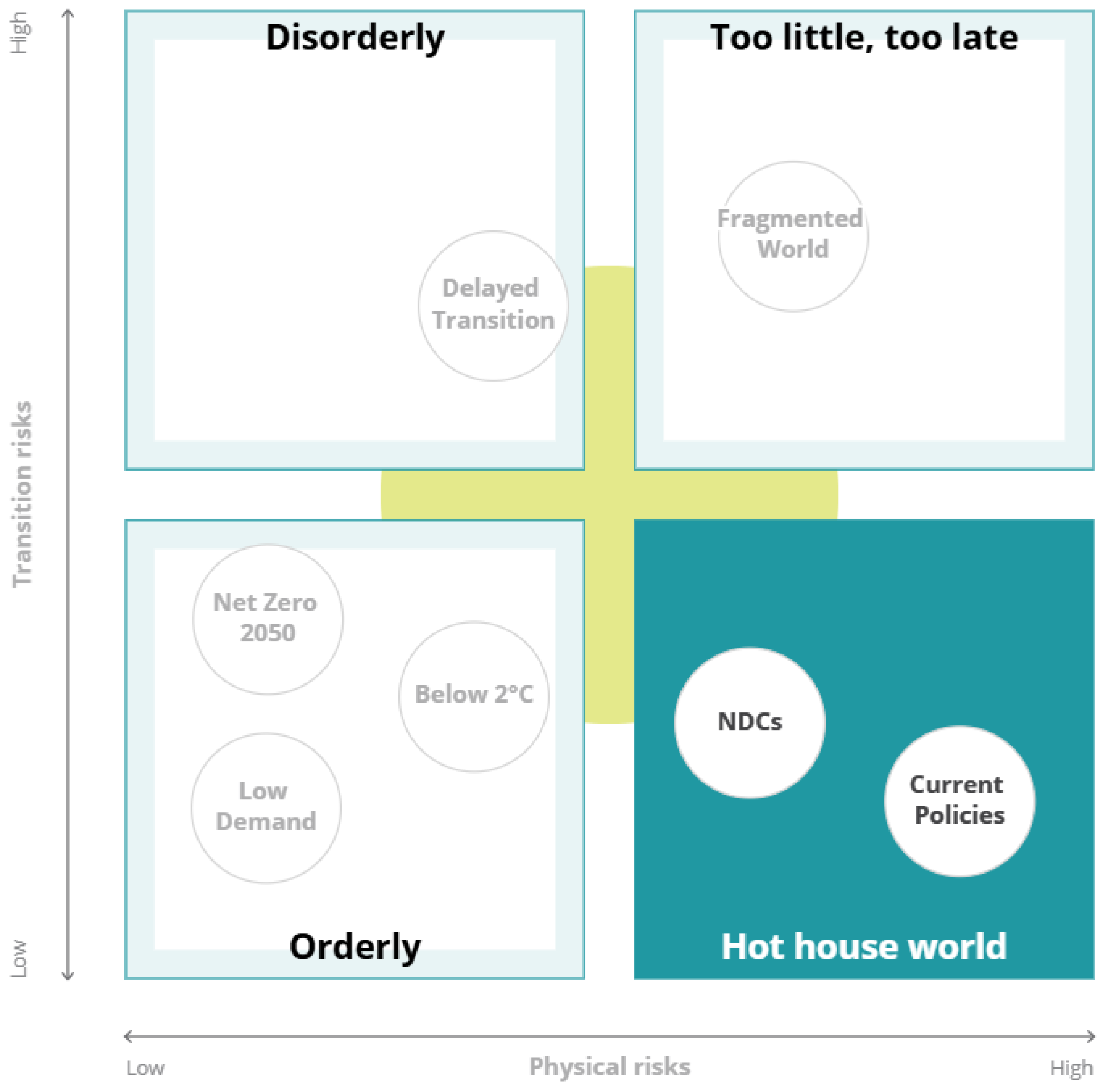

Supplementary Figure 5: Network for Greening the Financial System (NGFS) scenario narratives. *Figure depicts two elements of future climate-economy scenarios for use in financial institution analysis of climate change and the transition to a low-carbon economy: transition risks, arising from the decarbonisation of the economy; physical climate risks, arising from a warming world. Scenarios are grouped into four boxes: "Orderly", where climate policies are introduced relatively early, with lower resultant physical and transition risks, depending on the scenario; "Disorderly", where climate policies are delayed, leading to higher transition risks but relatively low physical risks; "Hot house world", where climate policies are insufficient to stem warming, leading to lower transition risks but higher physical risks; "Too little, too late", where a late and uncoordinated transition resulting in a fragmented (rather than collaborative) world leads to both high transition risk and high physical risk. Within these groupings, different scenarios describe particular social, economic and political dynamics. "NDC" = Nationally Determined Contributions, representing the current country pledges under the Paris Agreement, whereas Current Policies reflects climate policies that are actually in place. Reproduced from Ref[11], with permission from the NGFS secretariat.*

Simulate systemic risk dynamics

The simulation of how risks could occur, via system architectures, long-term stresses, triggers and crises, as well as the subsequent cascades and feedbacks once crises have occurred, can be undertaken in many ways. Based on the previous steps, detailed simulations can either be created through more advanced narratives, the specification of likelihoods (if relevant) around particular risk occurrences, perhaps through expert elicitations or (super)forecasts, and/or the development of

scenarios which could be quantified in different ways using modelling tools. An example is shown in Supplementary Fig. 6, which outlines simulated pathways of risks across biodiversity loss, developed using integrated assessment modelling[12].

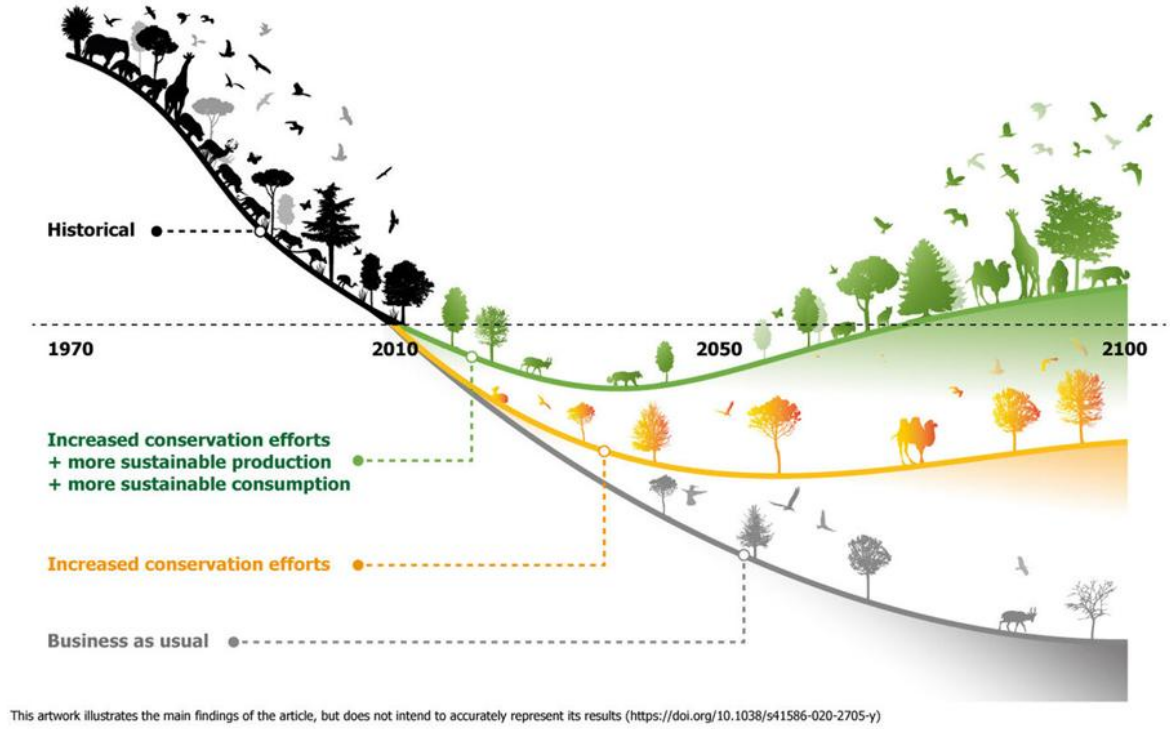

Supplementary Figure 6: Illustration of simulated scenarios around biodiversity loss, using integrated modelling *. Reproduction of figure based on analysis from Ref[12], with original figure production credit and copyright to Adam Islaam | International Institute for Applied Systems Analysis (IIASA).*

Implement, monitor, evaluate and adapt

Real-world systemic risk assessment and response exercises will require careful and ongoing monitoring of situations to understand risk and crisis evolution and the efficacy and other impacts of responses. These stages of the SRA process, which will trigger ongoing repetition of the preceding steps, would involve careful collection and monitoring of suitable metrics and evidence, around risks, crises, harms and responses. They will also require regular reviews of responses and plans, to understand where they may need adjustments or more fundamental changes in light of developments.

Apply cross-cutting practices at each stage

ASRA aims for its (and ultimately, society's) approach to systemic risk assessment to apply key principles[13] at each and every stage of the process. These principles inform a number of cross-cutting practices, as shown to apply across the assessment steps, as shown in Supplementary Fig. 1.

Participation of key stakeholders to ensure procedural and distributive justice means ensuring that those involved in and affected by systems under discussion and assessment are present and able to contribute to the SRA process. Consideration and representation of human AND non-human life and ecosystems means not simply undertaking SRA with the purpose of understanding risks and harms to people, but also to relevant ecosystems and their species. Cognitive diversity and trans-disciplinarity means engaging people from a wide variety of disciplines and backgrounds, and with a range of

perspectives, to help ensure all major facets of SRA are considered and included in the process. Using a diverse combination of quantitative and non-quantitative data, evidence and methods means actively avoiding over-reliance on any single source, approach or technique when undertaking SRA. For example, participatory systems mapping could be combined with qualitative narrative generation and quantitative modelling to estimate likelihoods or impacts of the materialisation of systemic risks. Data should be drawn from a wide variety of sources and evidence gaps actively identified, to stimulate enhanced future data and evidence gathering efforts. Data should be organised into a clear categorisation relating to systemic risk dynamics, including around system contexts, stresses, triggers and crises (see Supplementary Note 4). Red-teaming to address biases and blind spots and to stress-test assumptions means actively challenging assumptions at all stages, ideally through a formal incorporation of such red-teaming[14] processes, which involves deliberately adopting a critical thinking approach (often with a different set of people to those undertaking the main assessment) to challenge biases and assumptions, to improve processes and products. Recognition and communication of uncertainty means incorporating an understanding of the inherent uncertainty in many estimates of the size, or even direction, of relationships between factors and variables between systems. An example might be the response of different societal groups (decision-makers, publics, private organisations) to increases in greenhouse gas emissions – it could increase or decrease action on climate change in different contexts. Transparency and availability of data and methods means making assessment processes and their findings as clear and reproducible as possible, in line with FAIR (findable, accessible, interoperable and reusable) principles. In addition, ASRA's explicit focus on multiple ways of knowing, including via perspectives of Indigenous Peoples and Local Communities, requires CARE (Collective Benefit, Authority to Control, Responsibility, Ethics) principles. This means that wherever possible, but in collaboration with those providing data, particularly of a personal and sensitive nature, data and methods should be made explicit, available and accessible[15].

Summary checklist for framework

In every SRA procedure, the following questions (Supplementary Table 2) should be considered so as to address each of the steps in the process. At all stages the cross-cutting practices should also be considered.

Supplementary Table 2: Summary of key questions to consider at each stage of the framework

	**Key questions to consider**
**Detail systems architectures**	What are the goals of the system(s)?<br>Who do these systems serve?<br>What systems does it / do they closely connect to?<br>Why are we concerned about harms from these systems?
**Map systems interconnections**	What is the best way (given available time and resources) to map interconnections between systems?<br>What more could / should be done to capture interconnections in more detail?
**Identify existing, new and enhanced responses**	What are the critical leverage points of the system(s)?<br>What are the existing response types for the system(s) of focus?<br>What are potential enhanced and new response types?<br>How could the system(s) transform more fundamentally and what would be required?
**Assess response trade-offs and vulnerabilities**	What are the major trade-offs of envisaged responses?<br>Where are vulnerabilities in the responses, in light of future possibilities?
**Develop future storylines**	What are the scenarios in which the risks identified could propagate / subside, including consideration of response / no response storylines?<br>What are the implications of these scenarios across a variety of measures of systems?
**Simulate systemic risk dynamics**	Are there ways in which the storylines that are developed can be simulated analytically e.g. in models?<br>If applicable, what are the size of the adverse impacts / avoided impacts?<br>With what likelihood and confidence levels?
**Implement, monitor, evaluate, adapt**	How are risk and risk cascade dynamics developing?<br>How effective are responses?<br>What adaptations, revisions and course corrections are required?

Supplementary Note 3. Detailed analysis of food and energy system tools and approaches

Figure 3 in the main manuscript shows a categorisation of food and energy analytical tools, models and frameworks. A more detailed analysis of the key features of models is contained in a spreadsheet database (see Supplementary Data 1). For each tool, the analysis includes commentary on the following aspects, deemed relevant to the tool's applicability in systemic risk assessment:

- Description
- Sectors covered
- Time Horizon
- Scale / Geography
- Solution type
- Level of data required
- Links to socio-political factors
- Representation of non-linearities / shocks
- Links to other sectors
- Links from other sectors
- Extent of representation of any feedback loop dynamics

The catalogue contained within this database is not comprehensive, but aims to capture the main approaches utilised in analysis of food and energy systems, so as to provide an overview of the methods available and assist in identifying any gaps.

Supplementary Note 4. Data sources for supporting systemic risk analysis

As discussed in the main manuscript, there is likely to be a need to collect, collate and in some cases create new data sources relevant to the analysis of systemic risks within and across systems.

We categorise these data sources according to their relevance to each element of the creation, materialisation and transmission of risks as discussed in the main manuscript, namely according to the system context, the long-term stressors, the triggers that turn the resulting stressors into crises, and any responses to risks.

In more detail: **context** includes the economic, regulatory, environmental, social, and political conditions that shape vulnerabilities; **stressors** are persistent factors that erode resilience over time, while **triggers** are sudden events that precipitate **crises**. Crises result from the interplay of context, stressors, and triggers, leading to severe impacts within and/or across systems. Effective **response** involves systemic risk mitigation, preparedness, adaptation, and transformation.

This context-stressor-trigger-crisis-response (CSTCR) framing provides a structure within which to categorise data and evidence sources. For illustration, the following examples focus on food and energy systems, to reflect the focus of the main manuscript, with the addition of climate-relevant data sources, given the clear relevance of these to the food and energy systems analysis undertaken.

Climate

- Greenhouse gas (GHG) emissions data to track the **context** of rising emissions (e.g. Ref[16]).
- Temperature, precipitation, and sea level records to monitor **stressors** like climate change impacts (e.g. Ref[17]).
- Data on the frequency and severity of extreme weather events as potential crisis **triggers** (e.g. Ref[18]).

- Data on **crises** resulting from impacts of severe weather (e.g. humanitarian crises e.g. Ref[19])
- Policy **responses** to climate change such as adaptation policies (e.g. Ref[20])

Energy

- Data on energy production, consumption, and trade to understand the **context** and dependencies (e.g. Ref[21]).
- Infrastructure data on power plants, transmission grids, and pipelines to identify **stressors** (e.g. Ref[22])
- Cybersecurity incident data and threat intelligence to monitor potential **triggers** (e.g. Ref[23]).
- Market data on energy prices, volatility, and financial flows to detect instabilities and track **crisis** e.g. cost of living crisis, fuel poverty etc. (e.g. Ref[24])
- Data on energy policies, regulations, and investments to assess **response** measures (e.g. Ref[25]).

Agri-food

- Agricultural production and yield data to track the **context** and stressors like productivity trends (Ref[26]) .
- Data on land use, soil quality, water availability, and biodiversity to monitor key **stressors** (e.g. Ref[27]).
- Weather and climate data to identify potential **triggers** like droughts or floods (e.g. Ref[28]) .
- Food price, trade, and supply chain data to detect vulnerabilities, disruptions and emerging **crises** such as famine and food insecurity (e.g. Ref[29])
- Data on food security and access policy commitments, nutritional standards and food safety nets, to assess **response** capacities (e.g. Ref[30])

Supplementary Methods. Approach to determine SRA framework

Supplementary Figure 7 outlines the process undertaken over the period September 2023 to April 2024 to develop the ASRA systemic risk assessment (SRA) framework. Initial discussions held by a dedicated ASRA Working Group on systemic risk assessment (all co-authors on this study) determined that a “lens” of food system analysis would constitute a useful way of understanding intra- and inter-systemic risk links for a variety of systems. Further discussion determined that historical analysis of global-scale food systems crises would be an effective way of identifying salient factors and dynamics driving systemic crises. By focusing on a well-documented historical food crisis (of 2008), and a more recent crisis (of 2022, with dynamics still fresh in people’s minds), the analysis revealed that energy systems’ close coupling to food systems played a vital role in both crises. This led to an expansion of the analysis to encompass “entangled” food-energy crises over these periods.

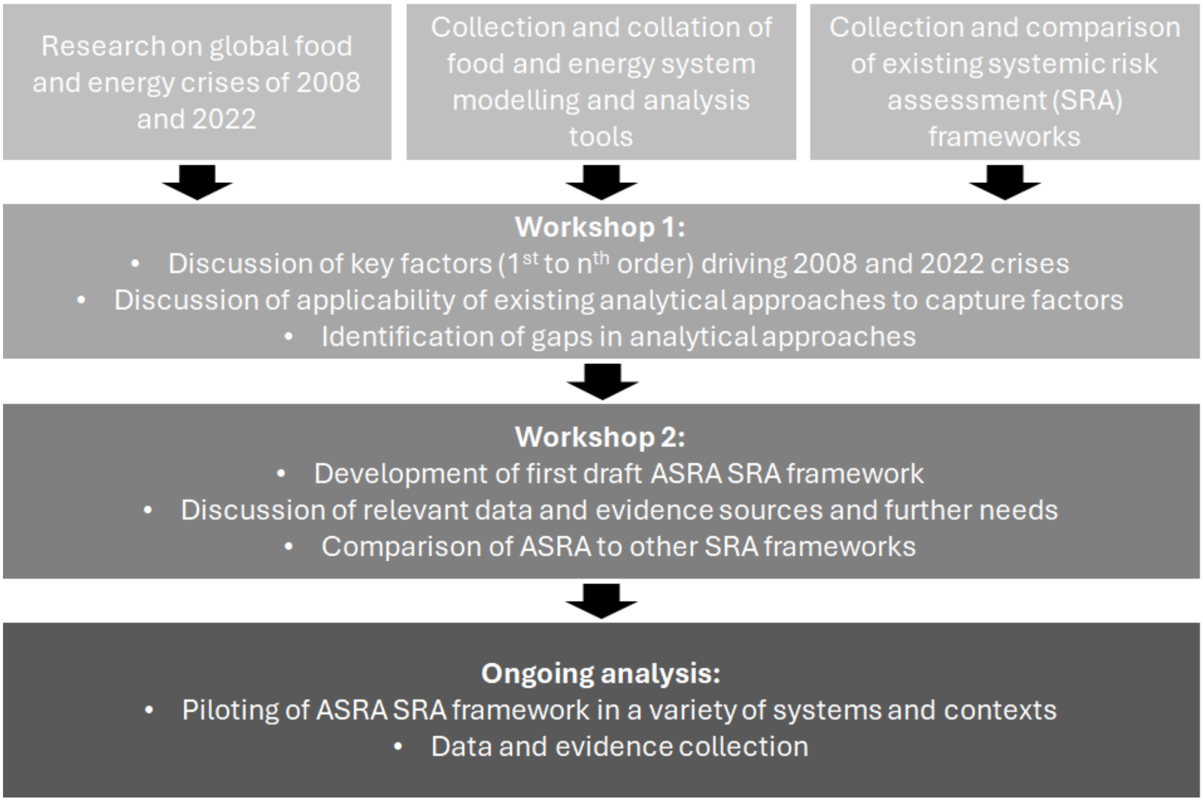

Supplementary Figure 7: Process for determining the ASRA SRA methodological framework. *This figure summarises the different research strands undertaken to arrive at the systemic risk assessment (SRA) methodological framework whose development is the focus of this study. Produced by authors.*

A literature review of key papers, studies and articles documenting these crises was then undertaken, as well as a review of the tools and models used to study food and energy systems. These initial investigations were accompanied by a review of existing methods and frameworks either designed specifically for, or clearly applicable to, systemic risk assessment, particularly when considering risks that can compound and/or cascade between systems.

This initial research set the context for two in-person discussions of the Working Group members, in January 2024. The Working Group first discussed the details of the specific historical food and energy crises, before focusing on a number of questions to help determine how to better assess such crisis episodes. Here “assess” is a deliberately broad term, encompassing aspects such as understanding risk drivers, risk transmission channels within and between systems, and risk likelihoods. As determined in the discussions that ensued, each and all of these has salience to the question of how

societies can better be prepared to assess and respond to such crises. The Working Group attendees split into two groups, to answer two pairs of questions over two 45-minute sessions. The first pair of questions asked:

- How do we identify the full range of relevant factors when thinking about the causes and consequences of these food-energy crises?
- How could we be confident we have considered the relevant factors and are not missing "surprise" factors?

The second pair of questions, designed to build directly on the first pair, asked:

- What approaches would allow inclusion of factors contributing to food-energy system crises AND the implications of these crises?
- How can such approaches be made tractable, given the complexity, data-heaviness and uncertainties involved?

A summary of each session's discussion points is shown in Supplementary Tables 3 and 4. As can be seen, there were overlaps in the discussion points between the two sessions, with shared themes including the need to define systems, their goals, the power and vulnerabilities within them, and the deep drivers of systemic risks. Also discussed across both sessions was the need for a diversity of perspectives, stakeholder views and modelling and analysis approaches, as well as clear communication, the need for new data and evidence, and the likelihood of trade-offs. Each of these elements has been directly translated into the ASRA SRA methodological framework.

Supplementary Table 3: Working Group discussion points on first pair of questions

Session 1 questions	*Key points raised*
• How do we identify the full range of relevant factors when thinking about the causes and consequences of these food-energy crises?<br><br>• How could we be confident we have considered the relevant factors and are not missing "surprise" factors?	• A critical first step is to articulate goals and work back from that.<br>• Need to consider whether risk analysis is about preserving a current, dysfunctional, system, or transforming it.<br>• Critical is to contextualise to local conditions.<br>• Identifying relevant factors requires cognitive diversity, with views from a range of experts. Important to ask different stakeholders what they consider to be a crisis, including critically the most vulnerable.<br>• Important to consider globalisation and concentration of food production systems. Local food resilience versus global food chains (including "big food", export practices, power dynamics).<br>• Useful to map out systems, to identify drivers of vulnerability and to identify feedbacks between factors.<br>• Important to consider that food crises trends are worsening, and why – including what we know and don't know.<br>• A system is also not just one thing – different levels of systems are different systems in and of themselves.<br>• Impossible to identify all factors, so relevant factors are key.<br>• Goal is not to eliminate uncertainty but to plan for it.<br>• Need to look beyond available information and in many cases create new information.<br>• Need to understand system lock-ins and vulnerabilities.<br>• Systems models, or multiple models, could be useful for stress testing, and to understand whether proposed responses are adequate.<br>• Important to consider diversity in views and approaches:<br>- A fully-comprehensive "digital twin" of the systems to be considered is unrealistic.<br>- Rather, a variety of tools and approaches should be used.<br>- Diversity in scenario and cases should also be considered.<br>• Temporal scales and scope of analysis should be made explicit<br>- This is important for considering the potential changes in power and values over different timescales.<br>- It's also important for making explicit short-term triggers, medium-term stresses and vulnerabilities, and longer-term process such as regime shifts.<br>• Red-Teaming and stress-testing of assumptions and identified factors is critical:<br>- Could convene an expert group with different / diverse views.<br>- Use "cruxing" to see where different approaches rely on particular drivers or assumptions to come to mostly similar results.<br>• Considering responses at the very outset is useful:<br>- This would, in combination with multi-stakeholder, participatory approaches, allow identification of no-regrets, bipartisan responses which are most likely to be implementable.

Supplementary Table 4: Working Group discussion points on second pair of questions

Session 2 questions	*Key points raised*
• What approaches would allow inclusion of factors contributing to food-energy system crises AND the implications of these crises?<br><br>• How can such approaches be made tractable, given the complexity, data-heaviness and uncertainties involved?	• In constructing approaches, it is worth being clear on the objective of the approach. This could be:<br>- General resilience building.<br>- Prediction.<br>- Identification of drivers and pathways.<br>- Crafting a response portfolio based on different assumptions and parameters.<br>- Identification of no-regrets strategies.<br>- Basic risk management.<br>• A suitable approach would enable identification of the deep systemic risks (e.g. water depletion, soil degradation) affecting food systems, as well as crisis triggers. This requires systemic stress, trigger and crisis analysis.<br>• In many cases we will not be assessing a system in and of itself, but rather its ability to reach identified goals.<br>- Some goals may be conflicting, but the trade-offs need to be highlighted early on.<br>- In many cases, addressing systemic risk is really identifying possibilities for systems change.<br>• Need a diversity of techniques and models rather than a “big beast” that does everything:<br>- Thinking about testing possible interventions to identify low/no-regret strategies is advisable in many cases (e.g. a Robust Decision Making approach).<br>- In addition, picking from a toolkit of approaches and using them to suit problem at hand and audience is appropriate – more so than building ever-more-complex models.<br>• To translate analysis into action, there are a number of considerations:<br>- How to fill the knowledge deficit.<br>- How to “persuade” – may need to apply toolkit to historical cases to show its worth, or may need to road-test it.<br>- How to speak to the “risk currency” of the audience i.e. their concerns and priorities.<br>- Be aware of response risks e.g. transition risks in climate change, nature and ecological responses.<br>• The data does not speak for itself, and the communication of risk and response can make or break any framework, analysis or model:<br>- Approaches will come under conflict and criticism, and important to make information and methods transparent and accessible.

Supplementary references

1. Mitchell, M. *Complexity: A Guided Tour*. (Oxford University Press, Oxford, 2011).
2. Zschau, J. Where are we with multihazards, multirisks assessment capacities? in (2017).
3. IEEE Reliability Society Technical Committee on 'Systems of Systems'. *Systems*. 5 (2014).
4. Centeno, M. A., Nag, M., Patterson, T. S., Shaver, A. & Windawi, A. J. The Emergence of Global Systemic Risk. *Annual Review of Sociology* **41**, 65–85 (2015).
5. Lawrence, M. *et al.* Global polycrisis: the causal mechanisms of crisis entanglement. *Global Sustainability* **7**, e6 (2024).
6. Lawrence, M., Shipman, M. & Homer-Dixon, T. *Introduction to Polycrisis Analysis*. 1–30 https://cascadeinstitute.org/technical-paper/introduction-to-polycrisis-analysis-guide/ (2024).
7. Barbrook-Johnson, P. & Penn, A. S. Introduction. in *Systems Mapping: How to build and use causal models of systems* (eds. Barbrook-Johnson, P. & Penn, A. S.) 1–19 (Springer International Publishing, Cham, 2022). doi:10.1007/978-3-031-01919-7_1.
8. Meadows, D. Leverage Points: Places to Intervene in a System. *The Academy for Systems Change* https://donellameadows.org/archives/leverage-points-places-to-intervene-in-a-system/.
9. ASRA. Systemic Risk Response Criteria. https://risk-assessment.transforms.svdcdn.com/production/assets/images/hero-v3.jpg?w=4000&h=2250&auto=compress%2Cformat&fit=crop&dm=1745921408&s=b385b6d1a9a8c74db0d54efe6e066c77 (2025).
10. Lempert, R. J. Robust Decision Making (RDM). in *Decision Making under Deep Uncertainty: From Theory to Practice* (eds. Marchau, V. A. W. J., Walker, W. E., Bloemen, P. J. T. M. & Popper, S. W.) 23–51 (Springer International Publishing, Cham, 2019). doi:10.1007/978-3-030-05252-2_2.
11. NGFS. NGFS Scenarios Portal. *NGFS Scenarios Portal* https://www.ngfs.net/ngfs-scenarios-portal/.
12. Leclère, D. *et al.* Bending the curve of terrestrial biodiversity needs an integrated strategy. *Nature* **585**, 551–556 (2020).

13. ASRA. *Principles for Systemic Risk Assessment and Response*. 1–5 https://www.asranetwork.org/insights/principles-for-systemic-risk (2024).
14. UK Ministry of Defence. Red Teaming Handbook. *GOV.UK* https://www.gov.uk/government/publications/a-guide-to-red-teaming (2021).
15. Carroll, S. R., Herczog, E., Hudson, M., Russell, K. & Stall, S. Operationalizing the CARE and FAIR Principles for Indigenous data futures. *Sci Data* **8**, 108 (2021).
16. UNFCCC. Greenhouse Gas Inventory Data - Time Series - Annex I. https://di.unfccc.int/time_series (n.d.).
17. The Inter-Sectoral Impact Model Intercomparison Project. *ISIMIP* https://www.isimip.org/.
18. Earth Science Data Systems, N. Environmental Justice Data Catalog | Earthdata. https://www.earthdata.nasa.gov/learn/environmental-justice-data-catalog (2022).
19. HDX. HDX Signals - Humanitarian Data Exchange. https://data.humdata.org/signals#data-coverage (n.d.).
20. European Union. Climate-ADAPT. https://climate-adapt.eea.europa.eu/en (n.d.).
21. EIA. International Energy Data. https://www.eia.gov/international/data/world (n.d.).
22. Gridfinder. Global energy infrastructure. https://gridfinder.rdrn.me/ (n.d.).
23. ENISA. Threat Landscape. *ENISA* https://www.enisa.europa.eu/topics/cyber-threats/threats-and-trends.
24. EIA. Petroleum & Other Liquids Data - U.S. Energy Information Administration (EIA). https://www.eia.gov/petroleum/data.php.
25. IEA. Policy database – Data & Statistics. *IEA* https://www.iea.org/policies (2020).
26. USDA. International Agricultural Productivity. https://www.ers.usda.gov/data-products/international-agricultural-productivity/ (n.d.).
27. Jian, J., Du, X. & Stewart, R. D. A database for global soil health assessment. *Sci Data* **7**, 16 (2020).

28. CRISP. CRISP: Climate Risk Planning & Managing Tool for Development Programmes in Agri-food Systems. https://crisp.eurac.edu/ (n.d.).

29. FEWS. Monitoring and forecasting acute food insecurity. https://fews.net/ (n.d.).

30. Economist Intelligence Unit. The Global Food Security Index. *UNCCD* https://www.unccd.int/resources/knowledge-sharing-system/global-food-security-index (n.d.).
