# Supplementary material for: A systemic risk assessment methodological framework for the global polycrisis
Source: Nat Commun. 2025 Aug 14;16:7382. doi: 10.1038/s41467-025-62029-w (PMC12354749; doi:10.1038/s41467-025-62029-w)
Supplement: Supplementary file 2 — Description of Addtional Supplementary Files [file 41467_2025_62029_MOESM2_ESM.pdf]

Description of Additional Supplementary File

Supplementary Data 1 - Detailed analysis of food and energy systems analytical approaches and models.
